# Urban food subsidies reduce natural food limitations and reproductive costs for a wetland bird

**DOI:** 10.1038/s41598-020-70934-x

**Published:** 2020-08-20

**Authors:** Betsy A. Evans, Dale E. Gawlik

**Affiliations:** 1 Department of Biological Sciences, Florida Atlantic University, 777 Glades Rd., Boca Raton, FL 33431 USA; 2 Environmental Science Program, Florida Atlantic University, 777 Glades Rd., Boca Raton, FL 33431 USA

**Keywords:** Zoology, Ecology, Environmental sciences, Ecology, Urban ecology, Wetlands ecology

## Abstract

There is a strong conservation need to understand traits of species that adapt to urban environments, but results have been equivocal. Wetland birds exhibit a strong phylogenetic signal towards urban tolerance; however, they have largely been ignored in urban studies. In their historic ranges, wetland birds inhabit dynamic systems, traveling long distances to locate food. This ability to exploit dynamic resources may translate to success in urban environments, areas characterized by novel food opportunities. We used the Wood Stork (*Mycteria americana*), a species of conservation concern, to determine if the ability to exploit resources in natural environments translated to exploitation of urban resources. During optimal natural foraging conditions, storks nesting in both urban and natural wetlands had narrow diet breadths and high productivity. However, during suboptimal conditions, urban stork diet expanded to include anthropogenic items, leading to increased productivity. Our study provides a mechanistic understanding of how a wetland species persists, and even thrives, in urban environments. We demonstrated that species inhabiting dynamic systems can exploit urban areas resulting in increased reproductive performance during suboptimal conditions. Together, urban environments may support biodiversity in a variety of ways, but species-specific mechanistic understanding will help highlight how to best mitigate potential threats of urbanization.

## Introduction

Urbanization is one of the most extreme forms of anthropogenic change impacting species globally^1,2^. Urbanization fragments and destroys natural habitats^2–6^, leading to increased pollution^7–9^, alteration of resources^5,10,11^ and species interactions^5,12,13^; all of which contribute to global biotic homogenization and biodiversity loss^2,14^. Nevertheless, recent studies show a more nuanced effect of urbanization, with urban areas increasingly being recognized as potential, vital components for biodiversity^15,16^, and in some cases, acting as refugia for species of conservation concern^17^.

The ability of some species to persist or even thrive in urban environments is largely associated with the ability to exploit resources and avoid risk in urban areas^18^. Consequently, as urbanization engulfs more of a landscape, it acts as a filter through which only some species can pass, resulting in more depauperate communities than surrounding areas^6^.

This ability to exploit resources is a key driver of the demographic response of avian species to urbanization; however, this response has been variable^5,11,19^. For example, in urban environments supplemental anthropogenic food can increase reproductive success whereas scarcity of natural food can reduce it^5,11,19^. Foraging theory predicts that as preferred resources become scarce^20^, lower value prey items should be added to the diet as individuals become more opportunistic and less selective^21^. When adults are feeding nestlings, one strategy to deal with unpredictable environmental conditions is to include different prey types in the diet^22^. Consequently, birds would be expected to access lower quality food when natural, higher quality food is less predictable or scarce.

There is a strong conservation need to understand the traits of native species that can move through the urban filter and adapt to these environments, but results have been equivocal. Successful invaders tend to have large clutch sizes, large brain size, and a generalist feeding strategy^23^. Moreover, there is a strong phylogenetic signal, with the waterbird clade having the highest degree of urbanization^23^. Despite a strong phylogenetic signal of urban tolerance among the waterbirds, less than 5% of studies have focused on these species (literature search using Web of Science and Google Scholar and the search terms ‘urban’ AND ‘bird’ AND ‘wetland’ OR ‘waterbird’).

The need to understand how native waterbirds are responding to urbanization is even more stark when considering the amount and types of wetlands that are created during the process of urbanization. In all but the most arid landscapes, drainage features are a prominent part of a developed landscape^24^. For example, new roads must have drainage features such as retention ponds, ditches, and canals, and new residential developments frequently must retain stormwater on-site in retention ponds. Created wetlands are now so common that a recent survey of coastal watersheds in the conterminous United States^25^ found that although freshwater wetlands showed a net loss, urban ponds increased by 19%, more than any other wetland type. Furthermore, of North American avifauna, wetland birds were the only avian clade to experience a net gain in numbers over the last several decades^26^.

The extent to which created wetlands may act as substitution habitats for native, wetland birds is not clear^27^. In their historic ranges, wetland birds occupy dynamic systems, which are characterized by resource pulses, driven through the inundation and drying of the landscape^28^. Wetland birds move large distances between habitats exploiting resources wherever they occur^29^, thus the ability to locate and exploit food resources over a large area is an important adaptation in these systems^30,31^. This flexibility in exploiting resources^29,31^ may translate to success in urban environments as these areas are characterized by novel food opportunities. In fact, substitution habitats are often characterized by abundant, predictable anthropogenic food sources^27,32^.

If native wetland birds are using urban resources and substituting urban habitats for natural wetlands, one of the most pressing questions is whether this process is beneficial or detrimental to wetland birds. We tested how the demographic response and diet of one such species, the Wood Stork (*Mycteria americana*; hereafter referred to as “stork”), differed between birds that nested in urban landscapes and natural wetlands. Storks provide a unique opportunity to study a wetland species that inhabits both a dynamic, natural system and an urban system. Storks have previously shown large scale changes in nesting patterns in response to decreases in food availability and suitable habitat driven by urban development^33–37^. The species was federally listed as Endangered in 1984^38^. However, by that time storks were expanding their nesting range northward^39^ and establishing colonies in or on the edge of urban areas^40^. The population size increased and in 2014 the stork was down-listed to Threatened^39^.

One of the key drivers of stork foraging success and nest initiation is food availability^35,41^. Food availability is highest when aquatic prey occur in high densities and are in shallow water where they are vulnerable to being captured. Unpredictable increases in water levels during the breeding season cause hydrologic reversals in the seasonal drying pattern, allowing prey to disperse out of drying pools thereby producing a marked drop in the density of prey per unit area and thus availability^42^. These reversals reduce prey availability for storks, which require certain water depths to forage optimally^43^. Hydrologic conditions can act as a proxy for wading bird food availability in the natural wetland system^28,44^. Since storks require specific foraging conditions to meet the increased caloric demands of nesting, food availability is considered a limiting factor^43^. What is unknown is if storks nesting in urban areas respond similarly to changes in natural wetland hydrologic conditions and food availability.

In order to determine the influence of natural and urban resources on nesting storks, we determined: (1) if productivity and body condition of stork nestlings differed between urban and natural wetland colonies; (2) if natural wetland hydrologic conditions and therefore natural wetland prey availability influenced the reproductive performance of both natural wetland and urban nesting storks; (3) if urban and natural wetland nesting storks increased their diet breadth in response to suboptimal hydrologic conditions and subsequently, lower prey availability; and (4) if stork diets resembled prey found in created wetlands during suboptimal natural wetland conditions. Based on foraging theory, we expected that diet breadth would increase during suboptimal hydrologic conditions, which are linked to low prey availability^43,45^. Furthermore, because urban storks have access to alternative food sources, we expected that urban birds would have greater diet breadth and productivity during suboptimal hydrologic conditions than storks nesting in natural wetlands. Overall, we provide strong evidence for the importance of urban resources for reproductive success in a wetland species of conservation concern.

## Results

We sampled 160 nests during the 2015–2017 nesting seasons. Of the 160 sampled nests, 106 nests were in three urban colonies and 54 nests were in two natural wetland colonies. We used 160 nests for productivity estimates and 114 nests for nestling body condition estimates (79 urban, 35 natural wetland; see Supplementary Table S1 online). There was no significant difference between the number of chicks produced in urban and natural wetland colonies (W = 2318.5, *P* = 0.062; see Supplementary Fig. S1 online); however, there was a significant difference in nestling body condition with slightly healthier chicks produced in natural wetland colonies (W = 1648.5, *P* = 0.041; see Supplementary Fig. S1 online). Hydrologic conditions influenced productivity, with significantly more chicks produced during optimal years than years with suboptimal and moderate conditions (χ^2^ = 14.29, *P* = 0.001; see Supplementary Fig. S1 online). Furthermore, significantly healthier chicks were produced during optimal than suboptimal conditions (W = 0.92, *P* = 0.00004; see Supplementary Fig. S1 online).

**Figure 1:**
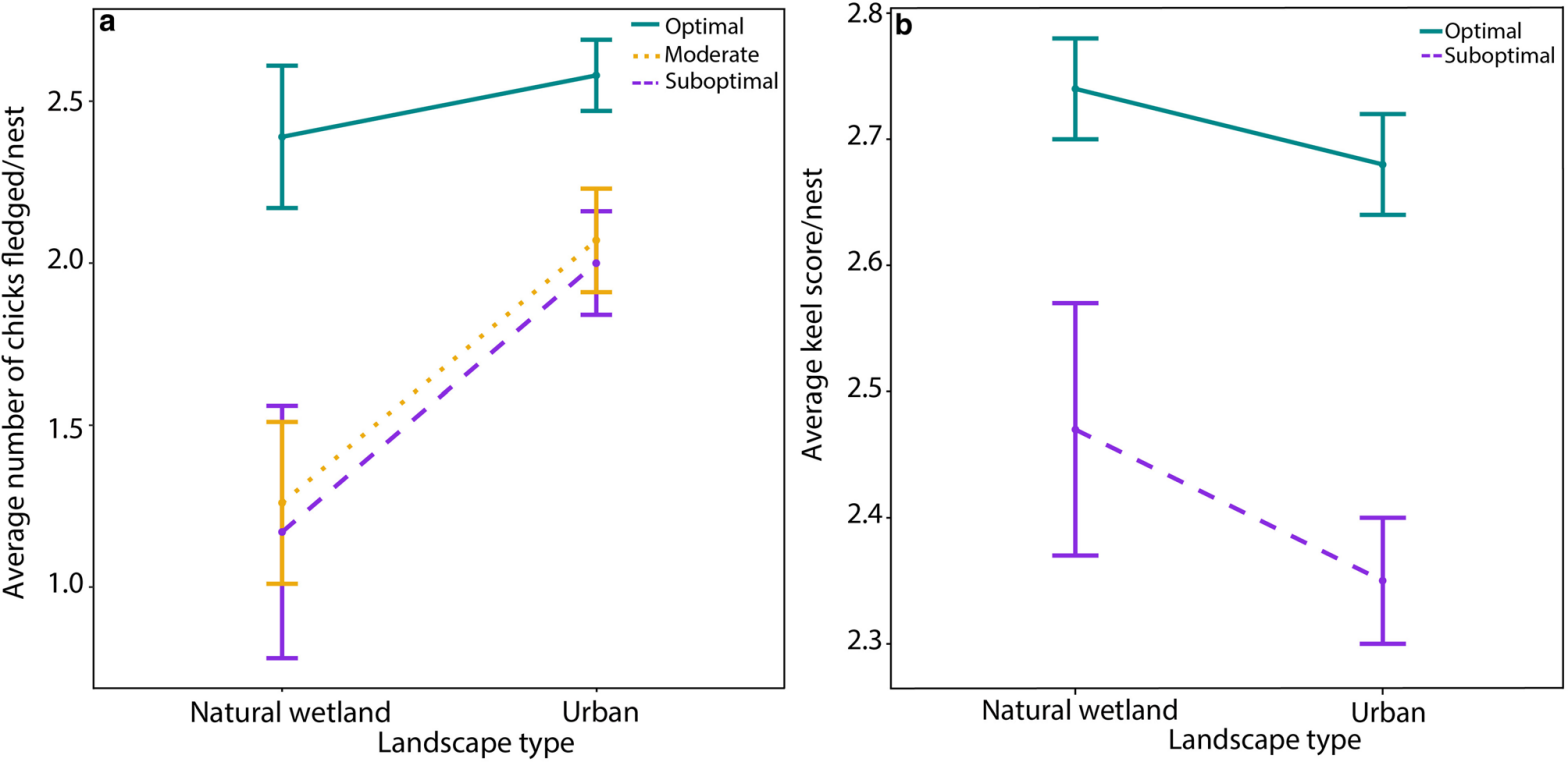
Productivity and body condition (mean ± SE) of storks across landscape type (urban and natural wetland) and hydrologic condition (suboptimal, moderate, optimal) in South Florida, 2015–2017. (**a**) Average number of chicks fledged per nest (productivity) across landscape type and hydrologic condition. (**b**) Average keel score per nest (body condition) across landscape type and hydrologic condition (suboptimal and optimal).

Productivity and body condition were influenced by both hydrologic conditions and landscape type (urban or natural wetland). The hydrologic condition model was the top model (w_i_ = 0.47, *R*^2^*m* = 0.14, *R*^2^*c* = 0.33; Table 1) explaining the variation in number of fledged chicks produced per nest. The second-best model was the landscape type/hydrologic condition model (w_i_ = 0.31, *R*^2^*m* = 0.18, *R*^2^*c* = 0.31; Table 1). The global model (including the interaction between hydrologic condition and landscape type) was the third-best model (w_i_ = 0.22, *R*^2^*m* = 0.24, *R*^2^*c* = 0.36; Table 1). Together, these models accounted for 100% of the Akaike weight. The coefficient for hydrologic condition had the strongest influence on productivity, with optimal conditions producing more chicks than suboptimal and moderate conditions (Table 2). For the hydrologic condition and landscape type interaction, the strongest influence on productivity was the interaction between urban landscape type and optimal hydrologic conditions, with urban colonies during optimal hydrologic conditions producing the most chicks (Fig. 1, Table 2). Similarly, natural wetland colonies in optimal years produced more chicks. Urban colonies in suboptimal and moderate hydrologic years produced more chicks than natural colonies; however, the confidence intervals minimally overlapped zero, so there is some uncertainty in this relationship (Fig. 1, Table 2).

**Table 1:** Results of generalized mixed models for productivity and body condition of storks, 2015–2017, South Florida.

Model	k	AIC_c_	∆AIC_c_	w_i_	*R*^2^*m*	*R*^2^*c*
**Productivity**						
Hydrologic condition	8	464.46	0.00	0.47	0.14	0.33
Landscape type/Hydrologic condition	10	465.28	0.82	0.31	0.18	0.31
Global	11	466.00	1.54	0.22	0.24	0.36
Null	5	472.82	18.10	0.00	0.00	0.16
Landscape type	7	483.37	18.91	0.00	0.04	0.15
**Body condition**						
Hydrologic condition	7	33.27	0.00	0.53	0.06	0.06
Landscape type/Hydrologic condition	9	34.24	0.96	0.33	0.06	0.06
Global	10	35.91	2.64	0.14	0.06	0.06
Landscape type	7	65.02	31.74	0.00	0.01	0.00
Null	5	66.10	32.84	0.00	0.00	0.00

Models are described with number of parameters (k), AIC_c_ values, differences in AIC_c_ values between the best model and each candidate model (∆AIC_c_), AIC_c_ weights (w_i_), the marginal R^2^ (*R*^2^*m* incorporates variance explained by fixed factors), and conditional R^2^ (*R*^2^*c*; incorporates variance explained by both fixed and random factors).

**Table 2:** Model averaged parameter estimates (β) for all models < 4 ∆AIC_c_ from the top model, 95% confidence limits (LCL, UCL), and variable importance values (Ʃw_i_) for models predicting productivity and body condition, 2015–2017, South Florida.

Parameter	β	LCL	UCL	Ʃw_i_
**Productivity**				
Intercept	**0.438**	0.057	0.854	1.00
Hydrologic condition				
Suboptimal	0			0.87
Moderate	− 0.023	− 0.241	0.295	0.87
Optimal	**0.364**	0.180	0.635	0.87
Landscape type				
Natural	0			0.38
Urban	0.234	− 0.133	0.610	0.38
Landscape type*Hydrologic condition				
Natural*Suboptimal	0			0.13
Urban*Suboptimal	0.328	− 0.221	0.876	0.13
Natural*Moderate	− 0.102	− 0.629	0.423	0.13
Urban*Moderate	0.376	− 0.179	0.931	0.13
Natural*Optimal	**0.543**	0.075	1.011	0.13
Urban*Optimal	**0.639**	0.102	1.177	0.13
**Body condition**				
Intercept	**0.877**	0.818	0.935	1.00
Hydrologic condition				
Suboptimal	0			0.86
Optimal	**0.130**	0.087	0.170	0.86
Landscape type				
Natural	0			0.33
Urban	− 0.033	− 0.085	0.020	0.33
Landscape type*Hydrologic condition				
Natural*Suboptimal	0			
Urban*Suboptimal	− 0.056	− 0.139	0.027	0.14
Natural*Optimal	**0.102**	0.025	0.178	0.14
Urban*Optimal	**0.080**	0.000	0.159	0.14

Bolded parameter estimate values indicate a parameter estimate where confidence intervals did not overlap zero.

Similar to the productivity models, the hydrologic condition model was the top model (w_i_ = 0.53, *R*^2^*m* = 0.06, *R*^2^*c* = 0.08; Table 1) explaining variation in body condition of nestlings. The landscape type/hydrologic condition model was the second-best model (w_i_ = 0.33, *R*^2^*m* = 0.06, *R*^2^*c* = 0.06; Table 1). The global model (including interaction between hydrologic condition and landscape type) was the third-best model (w_i_ = 0.14, *R*^2^*m* = 0.06, *R*^2^*c* = 0.06; Table 1). Together, these models accounted for 100% of the Akaike weight. Similar to the productivity parameter estimates, the hydrologic condition coefficient had the strongest influence on body condition, with optimal conditions producing healthier chicks than suboptimal conditions (Table 2). Chicks in urban colonies had lower body condition than chicks in natural wetland colonies; however, confidence intervals overlapped zero, so there is some uncertainty in this relationship (Fig. 1, Table 2). For the hydrologic condition and landscape type interaction, the healthiest chicks were produced during optimal hydrologic conditions in natural wetland colonies (Fig. 1, Table 2).

**Figure 2:**
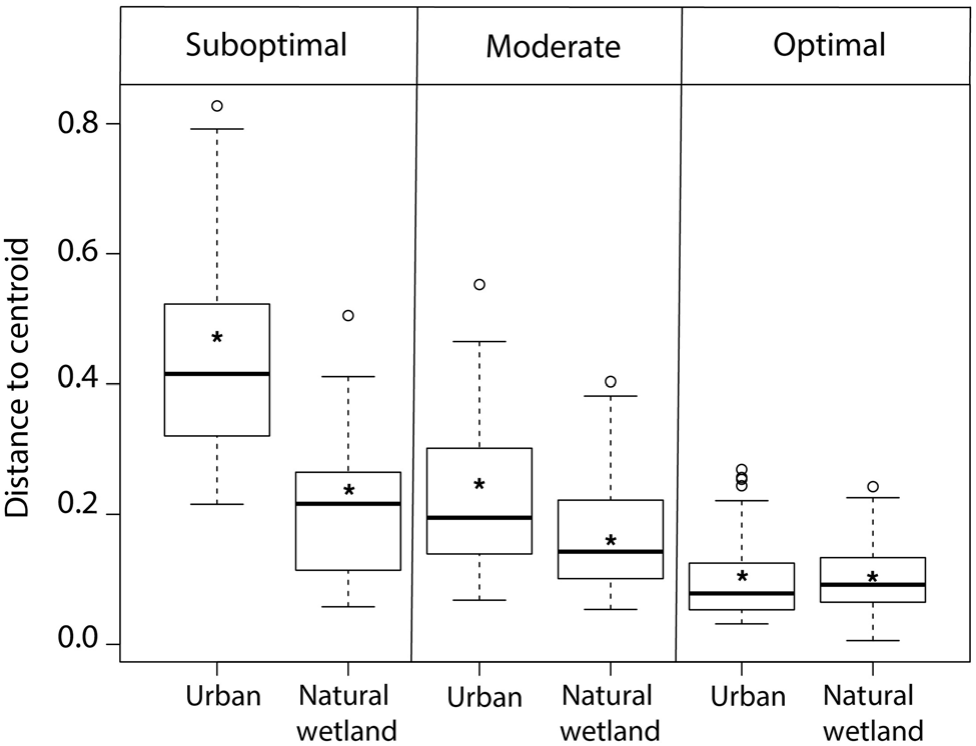
Variation in diet breadth of storks across landscape type (urban and natural wetland) and hydrologic condition (suboptimal, moderate, optimal), South Florida, 2015–2017. Diet breadth was determined as species dispersion in diet space using PERMDISP. A greater distance to centroid indicates a larger dispersion in diet. Within each box plot, the dark central horizontal line represents the median, the top and bottom horizontal lines are the first and third quartiles, respectively; and the whiskers are the range of values within 1.5 inter-quartile ranges of the first and third quartiles. Open circles represent outliers and asterisks represent mean diet breadth for each group.

**Figure 3:**
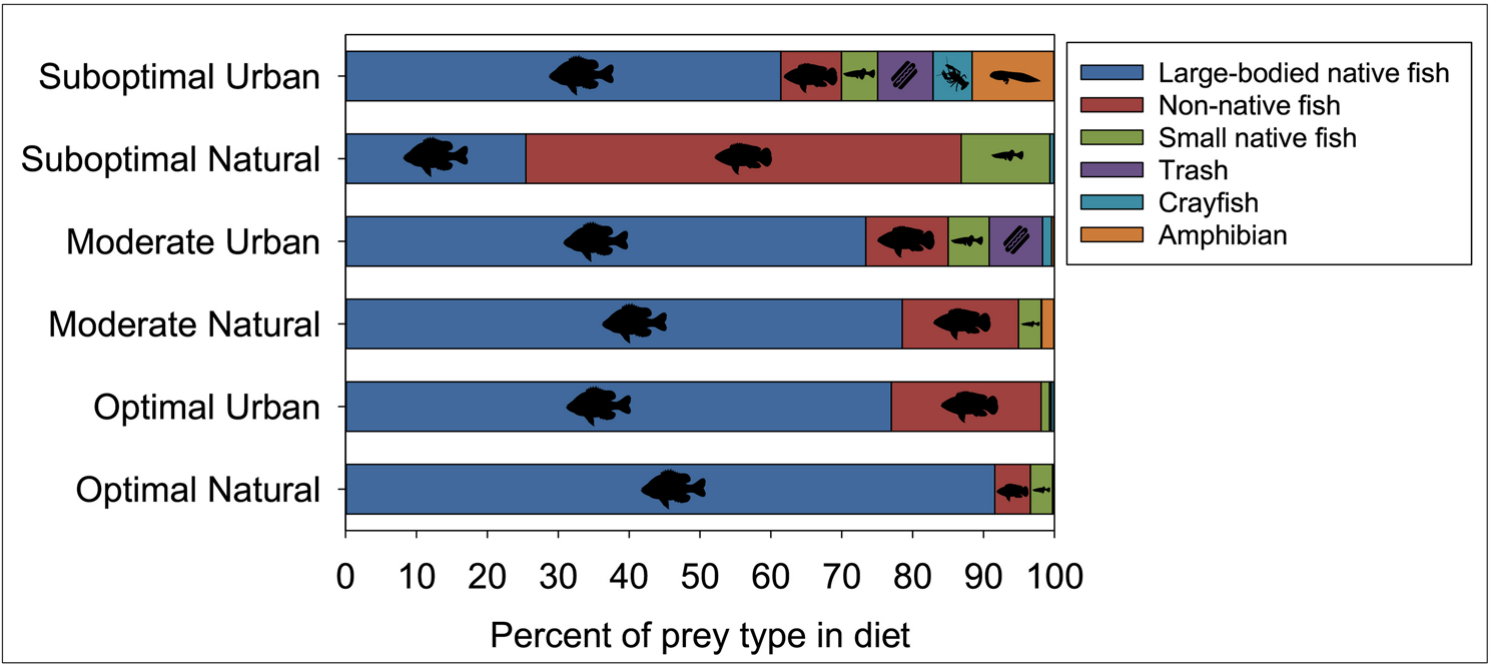
Percent of prey types within stork boluses (n = 643), South Florida, 2015–2017. See Supplementary Table S6 online for species or items that compose each prey type.

We collected 643 boluses from nestling storks during the 2015–2017 breeding seasons. Diet breadth differed with hydrologic conditions and landscape type (F_(5, 303)_ = 90.23, *P* = 0.001; Fig. 2). Diet breadth was greatest during suboptimal conditions in urban colonies whereas diet breadth constricted during optimal conditions in natural wetland colonies (Fig. 2). Diet breadth expanded during suboptimal conditions for both urban and natural wetland nesting birds and constricted during optimal conditions. During optimal years, diet breadth constriction resulted in no significant dietary difference between urban and natural wetland nesting birds (see Supplementary Table S2 online). Urban birds during suboptimal years expanded their diet to include a variety of different prey types such as amphibians (i.e., larval *Anura* species), trash, and crayfish whereas natural wetland birds primarily increased their consumption of non-native fish (Fig. 3). Overall, all storks during moderate and optimal conditions consumed large, native marsh fish, primarily sunfish (Fig. 3).

**Figure 4:**
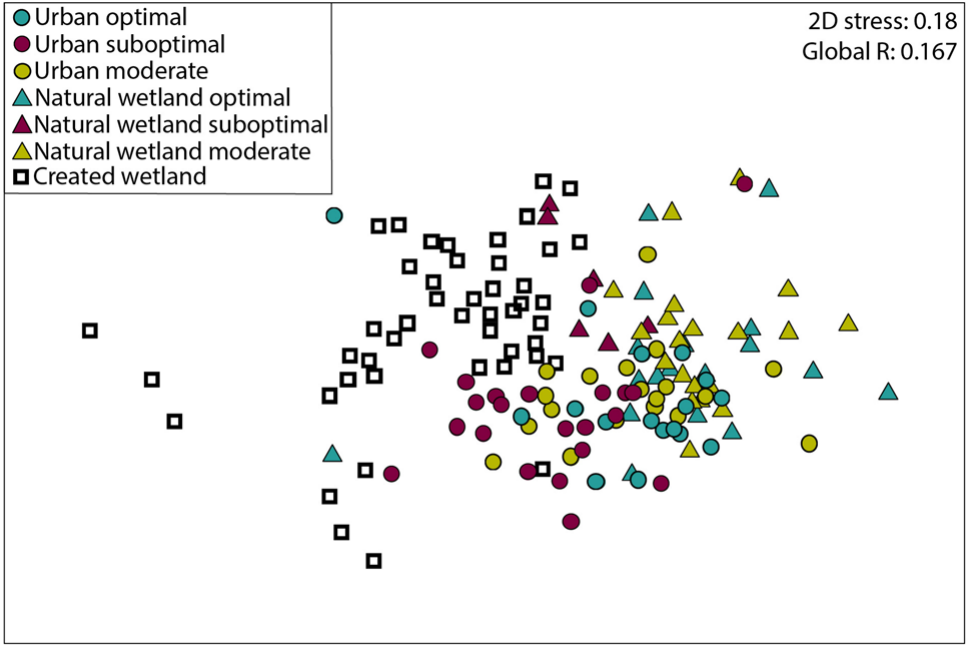
NMDS ordination of prey biomass depicting prey composition in created wetlands and stork boluses across hydrologic conditions and landscape type, South Florida, 2015–2017. Each bolus sample point is representative of prey composition within boluses collected in the same colony on the same date. Each created wetland sample point is representative of the prey composition found within created wetland sites each year. The proximity of points indicates the level of Bray–Curtis similarity in 2-dimensional space. Stress values indicate the degree of distortion relative to the actual multidimensional similarity between points. A stress value less than 0.20 indicates a useful 2-dimensional representation of the data.

Furthermore, prey composition of both urban (ANOSIM: R = 0.121) and natural wetland boluses (ANOSIM: R = 0.123) during suboptimal conditions had some overlap with created wetland prey composition whereas the greatest dissimilarity occurred during optimal (ANOSIM: natural wetland R = 0.364, urban R = 0.313) and moderate conditions (ANOSIM: natural wetland R = 0.399; urban R = 0.278; Fig. 4, see Supplementary Table S3 online).

Prey length (χ^2^ = 351.60, *P* = 0.00002) and weight (χ^2^ = 343.95, *P* = 0.00002) differed across hydrologic conditions and landscape type (see Supplementary Fig. S2, Table S4 online). Prey items were significantly smaller during suboptimal conditions and significantly larger during optimal and moderate conditions (see Supplementary Fig. S2 online). Furthermore, during optimal conditions, both prey length and weight were statistically similar in natural wetland and urban colonies; however, during suboptimal and moderate conditions prey length significantly differed between urban and natural wetland colonies (see Supplementary Fig. S2 online).

## Discussion

We demonstrated that wetland species can exploit urban areas when natural food resources are scarce. This ability to switch between habitats and thus resources allowed for better reproductive performance during periods of low natural food availability. Furthermore, body condition did not differ significantly between urban and natural wetland nesting birds during either optimal or suboptimal conditions, suggesting that supplemental anthropogenic resources do not negatively impact body condition. These findings indicate that urban areas can buffer a species from the unpredictability of natural food resources.

During suboptimal conditions, urban birds expanded their diets to include more prey types, including anthropogenic food, suggesting urban birds were able to exploit urban areas during low natural wetland prey availability. The ability of urban birds to switch their diet to include different prey types such as human-provided food (e.g., chicken wings, hotdogs) likely allowed them to produce more chicks during poor natural wetland prey availability conditions than their non-urban counterparts.

Not only were urban storks able to access human-provided food (i.e. trash), but they also increased the proportion of amphibians (i.e., larval *Anura* species) in their diet when natural wetland conditions were suboptimal. Larval Anurans occur in a wide range of wetland types in our study region; however, they were 10 times more abundant in roadside created wetlands (i.e. swales, ponds, canals) than in natural wetlands during the time period of this study^46^. This suggests that storks may also have been accessing created wetlands along roadways during suboptimal natural wetland conditions.

The demographic response of avian species to urbanization has been variable^5,11,19,47^. Wetland birds and other non-passerines, however, differ from passerines in their diet and habitat requirements, home range sizes, and reproductive ecology and may not be impacted by the same drivers. This likely explains our contrasting findings with many urban passerine studies which have observed negative reproductive responses to urbanization^11^. Proposed drivers of these negative responses include increased noise^48^ and light pollution^49^, predation^50,51^, and lower-quality habitat structure^52,53^. But importantly, there is little evidence that these factors impact the fitness of wetland species^34,54,55^.

A key difference in the response of wetland bird species to nesting in urban environments, is their ability to exploit adjacent habitats. For example, passerine species generally have smaller home range sizes than non-passerines^56^ and for the most part are spatially constrained and must rely on the resources found within a short distance of their nest. Wetland birds, on the other hand, often travel long distances to exploit resources in rapidly changing environments^29,31,41,57,58^.

Behavioural flexibility and the ability to travel long distances and exploit resources in dynamic systems may give wetland birds an ecological advantage in urban environments. During interpulse periods in natural wetlands, resource availability is low and waterbirds are often absent^29,43,58^; however, if a supplemental food source or subsidy become available, an animal can exploit this new resource^59^. Urban environments, in particular, are characterized by more predictable food sources^32^. This suggests that if urban areas are within the range of a species, these resources could be exploited. Our findings demonstrated that urban storks expanded their diets during times of low natural wetland prey availability to include resources commonly found in urban areas, partially diluting the natural wetland food limitation on wading bird populations^34,55^. Natural wetland birds, however, paid a greater reproductive penalty during suboptimal conditions than their urban counterparts. Furthermore, this ability to switch diets between resource pulses, may reduce population fluctuations and lower risk of extinction^59^.

Species in dynamic systems may cope with variable resources by being habitat and diet generalists, by dispersing away during resource poor periods, or by displaying a high degree of phenotypic plasticity whereby individuals’ preferred habitat or prey types change as resource availability changes^60–62^. Highly plastic species are predisposed to cope with the rapid nature of anthropogenic change and are more likely to succeed in altered landscapes than non-plastic species that cope via dispersal or positive adaptation^61–64^. Furthermore, rates of phenotypic change are greater in urbanized systems when compared with natural systems^65^. The ability of urban storks to switch between urban and non-urban environments may allow populations to respond quickly to the challenges of urbanization^61,66^. While these conclusions are based on a limited sample size of 3 years on one species, similar patterns are emerging globally which give us confidence in the findings. For example, the exploitation of resources in anthropogenic environments is not unique to storks in North America; other wetland species inhabiting dynamic systems have started to exploit anthropogenic resources worldwide. This ability to exploit alternative resources has allowed Marabou Storks (*Leptoptilos crumeniferus*) in Uganda to breed earlier^67^, White Storks (*Ciconia ciconia*) to alter home ranges and movements^68^, African Woolly-necked Storks (*Ciconia microscelis*)^69^ , Australian White Ibis (*Threskiornis molucca*)^70^ , and American White Ibis (*Eudocimus albus*) to inhabit urban areas^71,72^, and has even allowed Sacred Ibis (*Threskiornis aethiopicus*) to establish on new continents^73,74^.

## Conclusions

Urbanization continues to intensify with projected urban land cover expected to triple between 2000 and 2030^75,76^. Urbanization and human development have accelerated the loss of natural wetlands^77–79^, including their replacement by anthropogenic water features^25^. This degradation and replacement of natural wetlands suggests that urban areas may be imperative to wetland species, particularly when natural conditions are unpredictable. Our study provides evidence that created wetlands can act as substitution habitats and support a species of conservation concern when natural conditions are suboptimal.

Urban environments are increasingly being recognized as potential, vital components for biodiversity^15,16^, and in some cases, as refugia for species of conservation concern^17^.Furthermore, urban biodiversity and the presence of species of conservation concern can provide benefits to humans, such as improving human well-being and connecting people to nature^15^. Overall, we provide a mechanistic understanding of how a wetland species persists, and even thrives, in an urban environment. Wading birds, which often inhabit dynamic systems, have evolved a set of adaptations that allow them to be successful in urban environments through the ability to travel long distances and exploit resources both within and outside urban areas. We demonstrated that species inhabiting dynamic systems can exploit urban areas which result in increased reproductive performance during suboptimal natural system conditions. Together, urban environments may support biodiversity in a variety of ways, but species-specific mechanistic understanding will help highlight how to best mitigate the potential threats of urbanization.

## Methods

### Study area

**Figure 5:**
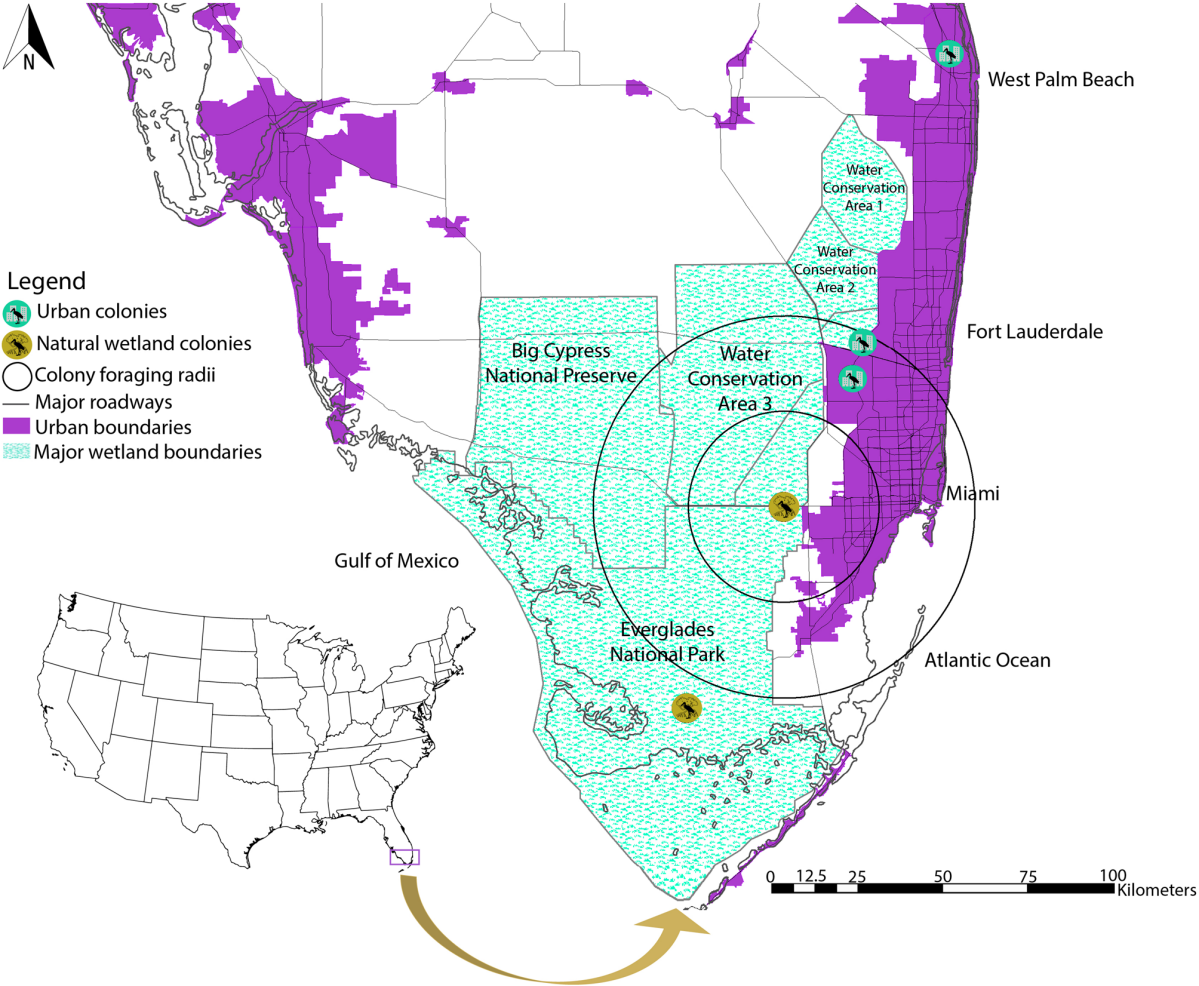
Location of stork nesting colonies (n = 5) in South Florida, 2015–2017. Colony foraging radii represents the typical minimum (25 km) and maximum (50 km) stork foraging distances^100^. Locations of three urban colonies: Griffin (26°3′48.95″N, 80°21′59.30″W), Sawgrass (26°8′59.31″N, 80°20′15.69″W), BallenIsles (26°49′48.34″N, 80°6′33.17″W); Locations of two natural wetland colonies located in Everglades National Park: Paurotis Pond (25°16′54.39″N, 80°48′5.09″W), Tamiami West (25°45′32.43″N, 80°32′42.22″W). We did not collect boluses from Tamiami West in 2016 as no storks nested in the colony, likely due to suboptimal hydrologic conditions in the natural wetland system. Map generated in ESRI ArcMap 10.4.1 (https://www.esri.com)^101^.

We conducted our study in South Florida where a vast freshwater wetland, the Everglades, is located adjacent to a large urban area (Fig. 5). Due to its expansive size, the Everglades includes several wetland habitat types with distinct vegetative communities^80^. Common marsh vegetation includes sawgrass (*Cladium jamaicense*), spikerush (*Eleocharis* spp.), maidencane (*Panicum hemitomon*), bladderwort (*Utricularia* spp.) and water lily (*Nymphaea odorata*). Tree island communities, where storks nest, are dominated by willow (*Salix* spp.), cypress (*Taxodium* spp.), and pond apple (*Annona glabra*). Water control structures, such as canals and levees, separate the Everglades into Water Conservation Areas to the north and Everglades National Park to the south. Study sites located within urban South Florida included similar native marsh and tree island community vegetation in addition to non-native vegetation such as hydrilla (*Hydrilla verticillata*), water hyacinth (*Eichhornia crassipes*), water lettuce (*Pistia stratiotes*), Brazilian pepper (*Schinus terebinthifolius*), and Australian pine (*Casuarina* spp.).

### Natural wetland hydrologic conditions and prey availability

The relationship between natural wetland hydrologic conditions, prey availability, and wading bird populations is well-studied in the Florida Everglades^33–35,41,43,55^. Known hydrologic conditions that impact stork prey availability include water depth, recession rate, days since drydown, and water level reversals^81,82^. Thus, hydrologic conditions can act as a proxy for wading bird food availability in the natural wetland system^28,44^.

Hydrologic conditions were variable during the study period. 2015 began with moderate water levels with a steady water level recession; however, there were hydrologic reversals throughout the season. In 2016 South Florida experienced unseasonably high rainfall with minimal recession, whereas in 2017 the region had below average dry season rainfall, allowing for a steady water level recession throughout the dry season.

To confirm that hydrologic conditions were related to natural wetland foraging for storks, we calculated available foraging habitat for wading birds using the Everglades Depth Estimation Network (EDEN), a real-time hydrologic monitoring network that provides daily water depths at a 400 × 400 m scale for the Everglades^83^. If a previous wet cell dried to a depth of 0 during the breeding season, that cell contained water that was shallow enough for foraging at some point during the dry season. EDEN water depths are derived from a single elevation at the centre of the 400 × 400 m cell, thus when a cell has a water depth of 0 cm, portions of the cell may have standing water. In 2015, a moderate year, 57% of the natural wetland system was available to foraging storks. In 2016, a suboptimal year, 36% was available for foraging, and in 2017, an optimal year, 81% of the system was available for foraging (see Supplementary Fig. S3 online).

### Field methods

We visited two natural wetland colonies and three urban colonies 1–2 times per week during the 2015–2017 breeding seasons (approximately March through June; Fig. 5). We selected these study colonies based on their range of hydrological conditions and history of repeated use by nesting storks. We describe colony landscape type broadly as either “urban” or “natural wetland” with natural wetland colonies occurring within Everglades National Park and urban colonies occurring within the urban east coast corridor of South Florida. At each colony location, we marked individual nests from which we collected productivity, body condition, and diet information.

#### Productivity and body condition

To assess reproductive performance of urban and natural nesting storks, we measured productivity and nestling body condition. To determine productivity, we recorded the number of chicks fledged per nest at 4 weeks. Rodgers^84^ determined fledgling age at 7 weeks; however, nestlings in urban colonies often moved among nests after 4 weeks of age due to many nests being crowded into small created islands. Thus, to directly compare urban and natural wetland colonies we determined fledgling to be at 4 weeks.

To assess the body condition of nestlings, we used a score for pectoral muscle mass. Similar studies in the Everglades have found that pectoral muscle mass scores were an effective indicator of body condition for wading bird nestlings^85^. We scaled keel scores from 1 to 5 with 1 having a prominent keel and 5 having muscle greater than flush with the keel. We only monitored body condition of nestlings during suboptimal (2016) and optimal (2017) hydrologic conditions. Keel scores were averaged for each nest at the age of fledging (4 weeks).

Additionally, we explored the potential relationship of brood size and clutch size on body condition. Clutch size remained relatively constant between suboptimal and optimal years and brood size was not strongly correlated to nestling body condition (see Supplementary methods and Table S5 online), thus these variables were not included in body condition models.

#### Diet breadth

To determine if diet breadth differed with hydrologic conditions, we collected food boluses (stomach regurgitations) from nestlings. Nestlings often regurgitate in the presence of humans, making bolus contents readily available for collection (see Supplementary Methods online). We identified all prey to the lowest possible taxonomic level (usually species), weighed to the nearest 0.01 g, measured to the nearest mm, and noted whether each piece represented a partial or whole prey species. We grouped species into prey type categories based on habitat, size, and taxonomic grouping. The categories were small native marsh fish, large-bodied native marsh fish, non-native fish, crayfish, amphibians, and trash (see Supplementary Table S6 online). We recorded both total and standard length for fish species, and total length for invertebrates.

#### Created wetland prey composition

To determine if diets resembled prey found within created wetlands, we sampled four created wetland types: swales, canals, permanently inundated stormwater ponds, and ephemeral stormwater ponds. We randomly identified created wetlands from a digitized map and selected them after to determine whether they were accessible to foraging storks. Created wetland sites were located along major roadways in the east and west coast urban corridors, Water Conservation Areas, and Big Cypress National Preserve (Fig. 5). A total of 36 sites evenly dispersed across the urban corridors, Water Conservation Areas, and Big Cypress National Preserve were sampled monthly in 2015 and 2016.

For samples, we transferred captured fauna directly to jars containing a solution of water and tricaine methanesulfonate (MS 222; 300 mg/L). Similar to our bolus samples, we identified all prey to the lowest possible taxonomic level (usually species), weighed to the nearest 0.01 g, and measured to the nearest mm. We recorded both total and standard length for fish species, and total length for invertebrates. Full details of field method sampling are provided in Supplementary Methods online.

### Statistical methods

#### Productivity and body condition

We used the nonparametric Wilcoxon Rank Sum test to compare productivity and body condition between landscape types. We compared productivity among hydrologic conditions, using the nonparametric Kruskal–Wallis test and used the Wilcoxon Rank Sum test for body condition. We used non-parametric tests due to unequal sample sizes and uneven variance among groups with non-normal distributions.

To investigate the potential interaction between landscape type and hydrologic conditions, we used generalized linear mixed models (GLMMs) as data were not normally distributed and we could not use parametric approaches, such as a two-way crossed ANOVA. We used an information-theoretic approach to investigate how landscape type and hydrologic conditions affect productivity and body condition of storks^86^. We developed a priori candidate models for both body condition and productivity (see Supplementary Table S7 online). We had four competing models for determining productivity and body condition of nestling storks.

Urban passerine birds generally have fewer chicks fledged per nest and lower body condition of nestlings^11^. Thus, the “landscape type hypothesis” predicts that urban colonies will have lower productivity and overall body condition of chicks regardless of hydrologic conditions. However, since wading birds in the Everglades are food-limited^43^, we proposed the “hydrologic condition hypothesis” where we would expect that productivity and body condition would vary with natural wetland conditions. Thus, we predict that during suboptimal years when foraging habitat and prey availability is low, productivity and body condition would be lowest. We also included models that investigated the effects of both landscape type and hydrologic conditions on productivity and body condition.

All model analyses were conducted in R 3.4.2^87^. We used Akaike’s Information Criterion for small sample sizes (AIC_c_) to determine which of our a priori models were most parsimonious (see Supplementary Table S7 online). We calculated ΔAIC_c_ values and model probabilities (w_i_) to determine the distance between the best model and other models in the candidate set. To determine the importance of individual explanatory variables, we calculated summed Akaike weights (Ʃw_i_) for each model containing the variable. We calculated averaged parameter estimates for all models < 4 ∆AIC_c_ from the top model to determine the effect of an explanatory variable on either the number of chicks fledged per nest (productivity) or the average keel score per nest (body condition). To examine model variability, we also calculated 95% confidence intervals for the model-averaged parameter estimates. Lastly, we calculated a marginal and conditional correlation coefficient for each model to evaluate model fit. The marginal coefficient (*R*^2^*m*) represents the variance explained by fixed effects whereas the conditional coefficient (*R*^2^*c*) represents the variance explained by both random and fixed effects^88^.

To determine the effect of landscape type and hydrologic conditions on productivity, we used a Conway-Maxwell-Poisson distribution for count data. The Conway-Maxwell-Poisson is a flexible distribution that accounts for under-dispersed data and is generally a good fit for count data, such as clutch or brood size^89,90^ (see Supplementary Fig. S4-S6 for model diagnostics online). We included colony (n = 5) as a random effect to control for any variation within landscape type (urban or natural wetland). Colony met the minimum requirement of five levels for a random effect^91^. Generalized linear mixed models were built using the R package “glmmTMB”^92^ and model diagnostics were conducted in the R package “DHARMa”^93^.

To determine the effect of landscape type and hydrologic conditions on body condition, we fit the model with a Gaussian error and a log-link. Body condition generalized linear mixed models were built using the package “lme4”^94^ and model diagnostics were conducted in the package “DHARMa”^93^ (see Supplementary Fig. S7–S9 for model diagnostics online).

#### Diet breadth

To determine how prey composition and diet breadth differed between landscape types and with hydrologic conditions, we used nonparametric multivariate techniques. We calculated total biomass of each prey species found within boluses collected and eliminated prey species that accounted for less than 1% of total biomass to prevent over representation of rare species. We transformed the data using a square-root transformation which allowed for a greater contribution of rare species^95^.

We used PERMANOVA to examine differences in prey composition across hydrologic conditions and landscape type^96^. P values for the pseudo-F statistic were based on 999 permutations. Colony was included as a random factor to account for variation in landscape type (urban or natural). We performed a test for homogeneity of multivariate dispersion (PERMDISP)^97^ to determine if diet breadth differed among landscape type and hydrologic conditions groups (i.e. suboptimal urban, suboptimal natural). Differences in diet breadth were measured as dispersion in diet space. PERMDISP measured the distances of each individual bolus sample to the hydrologic condition and landscape type group multivariate median (i.e. centroid) and assessed differences in dispersion among groups to indicate which groups have a more restricted diet than other groups. All analyses were conducted in R 3.4.2^87^. PERMANOVA and PERMDISP analyses were conducted in R using the vegan package^98^.

To determine if diets resembled prey composition of created wetlands, we used non-metric multi-dimensional scaling (NMDS) derived from Bray–Curtis similarities, using PRIMER-E^99^. We used NMDS plots to examine the similarity or dissimilarity of prey composition between stork diets and created wetlands. We used an ANOSIM analysis to determine if there was a statistical difference between prey composition of diet samples and created wetlands during suboptimal conditions.

We further examined differences in prey composition by comparing prey length and weight among hydrologic conditions and landscape types, using the nonparametric Kruskal–Wallis test. We used non-parametric tests due to unequal sample sizes and uneven variance among groups with non-normal distributions. All analyses were conducted in R 3.4.2^87^. Results were considered significant when P values were below 0.05.

### Ethics statement

All experimental protocols were approved by the following institutions and federal and state agencies: Florida Atlantic University's Institutional Animal Care and Use Committee (A14-11, A14-28), Big Cypress National Preserve (BICY-2014-SCI-0014), Everglades National Park (EVER-2014-SCI-0021, EVER-2016-SCI-0021), Florida Fish and Wildlife Conservation Commission (S-15-02), and United States Fish and Wildlife Service (TE65550A-0). All methods were carried out in accordance with relevant guidelines and regulations.

## Supplementary information

Supplementary information.

## Data Availability

The data and materials used in this manuscript are available through request to the corresponding author.

## References

[1] McKinney ML (2002). Urbanization, biodiversity, and conservation. Bioscience.

[2] McKinney ML (2006). Urbanization as a major cause of biotic homogenization. Biol. Conserv..

[3] Marzluff JM, Ewing K (2001). Restoration of fragmented landscapes for the conservation of birds: a general framework and specific recommendations for urbanizing landscapes. Restor. Ecol..

[4] Fahrig L (2003). Effects of habitat fragmentation on biodiversity. Annu. Rev. Ecol. Evol. Syst..

[5] Chace JF, Walsh JJ (2006). Urban effects on native avifauna: a review. Landsc. Urban Plan..

[6] Shanahan DF, Strohbach MW, Warren PS, Fuller RA, Gil D, Brumm H (2014). The challenges of urban living. Avian Urban Ecology: Behavioural and Physiological Adaptations.

[7] Longcore T, Rich C (2004). Ecological light pollution. Front. Ecol. Environ..

[8] Patricelli GL, Blickley JL (2006). Avian communication in urban noise: causes and consequences of vocal adjustment. Auk.

[9] Grimm NB (2008). The changing landscape: ecosystem responses to urbanization and pollution across climatic and societal gradients. Front. Ecol. Environ..

[10] Fuller RA, Warren PH, Armsworth PR, Barbosa O, Gaston KJ (2008). Garden bird feeding predicts the structure of urban avian assemblages. Divers. Distrib..

[11] Chamberlain DE (2009). Avian productivity in urban landscapes: a review and meta-analysis. Ibis.

[12] Faeth SH, Warren PS, Shochat E, Marussich WA (2005). Trophic dynamics in urban communities. Bioscience.

[13] Shochat E (2010). Invasion, competition, and biodiversity loss in urban ecosystems. Bioscience.

[14] Blair RB (1996). Land use and avian species diversity along an urban gradient. Ecol. Appl..

[15] Dearborn DC, Kark S (2010). Motivations for conserving urban biodiversity. Conserv. Biol..

[16] Callaghan CT (2019). Heterogeneous urban green areas are bird diversity hotspots: insights using continental-scale citizen science data. Landsc. Ecol..

[17] Ives CD (2015). Cities are hotspots for threatened species. Glob. Ecol..

[18] Sol D, González-Lagos C, Moreira D, Maspons J, Lapiedra O (2014). Urbanisation tolerance and the loss of avian diversity. Ecol. Lett..

[19] Kettel EF, Gentle LK, Quinn JL, Yarnell W (2018). The breeding performance of raptors in urban landscapes: a review and meta-analysis. J. Ornithol..

[20] Stephens DW, Krebs JR (1986). Foraging Theory.

[21] MacArthur RH, Pianka ER (1966). On optimal use of a patchy environment. Am. Nat..

[22] Wright J, Both C, Cotton PA, Bryant D (1998). Quality vs. quantity: energetic and nutritional trade-offs in parent provisioning strategies. J. Anim. Ecol..

[23] Callaghan CT (2019). Generalists are the most urban-tolerant of birds: a phylogenetically controlled analysis of ecological and life history traits using a novel continuous measure of bird responses to urbanization. Oikos.

[24] Butler D, Digman CJ, Makropoulos C, Davies JW (2018). Urban Drainage.

[25] Dahl, T. E. & Steadman, S. M. Status and trends of wetlands in the coastal watersheds of the Conterminous United States 2004 to 2009. U.S. Department of the Interior, Fish and Wildlife Service and National Oceanic and Atmospheric Administration, National Marine Fisheries Service, Washington D.C. 46 pp. (2013).

[26] Rosenberg KV (2019). Decline of North American avifauna. Science.

[27] Martínez-Abraín A, Jiménez J (2016). Anthropogenic areas as incidental substitutes for original habitat. Conserv. Biol..

[28] Botson BA, Gawlik DE, Trexler JC (2016). Mechanisms that generate resource pulses in a fluctuating wetland. PLoS ONE.

[29] Kingsford RT, Roshier DA, Porter JL (2010). Australian waterbirds—time and space travellers in dynamic desert landscapes. Mar. Freshw. Res..

[30] Kingsford RT, Curtin AL, Porter J (1999). Water flows on Cooper Creek in arid Australia determine ‘boom’ and ‘bust’ periods for waterbirds. Biol. Conserv..

[31] Roshier DA, Whetton PH, Allan RJ, Robertson AI (2001). Distribution and persistence of temporary wetlands in arid Australia in relation to climate. Austral Ecol..

[32] Oro D, Genovart M, Tavecchia G, Fowler MS, Martínez-Abraín A (2013). Ecological and evolutionary implications of food subsidies from humans. Ecol. Lett..

[33] Kushlan JA, Frohring PC (1986). The history of the southern Florida Wood Stork population. Wilson Bull..

[34] Frederick PC, Spalding MG, Davis SM, Ogden JC (1994). Factors affecting reproductive success of wading birds (Ciconiiformes) in the Everglades ecosystem. Everglades: The Ecosystem and its Restoration.

[35] Ogden JC, Davis SM, Ogden JC (1994). A comparison of wading bird nesting dynamics, 1931–1946 and 1974–1989 as an indication of changes in ecosystem conditions in the southern Everglades. Everglades: The Ecosystem and Its Restoration.

[36] Crozier GE, Gawlik DE (2003). Wading bird nesting effort as an index to wetland ecosystem integrity. Waterbirds.

[37] Frederick P, Gawlik DE, Ogden JC, Cook MI, Lusk M (2009). The white ibis and wood stork as indicators for restoration of the everglades system. Ecol. Indic..

[38] United States Fish and Wildlife Service (USFWS) (1996). Revised recovery plan for the U.S. breeding population of the wood stork.

[39] United States Fish and Wildlife Service (USFWS) (2014). Reclassification of the U.S. breeding population of the Wood Stork from endangered to threatened. Federal Regist..

[40] Gawlik DE (2000). South Florida Wading Bird Report.

[41] Kahl MP (1964). Food ecology of the wood stork (*Mycteria americana*) in Florida. Ecol. Monogr..

[42] Yurek S, DeAngelis DL (2019). Resource concentration mechanisms facilitate foraging success in simulations of a pulsed oligotrophic wetland. Landsc. Ecol..

[43] Gawlik DE (2002). The effects of prey availability on the numerical response of wading birds. Ecol. Monogr..

[44] Trexler JC, Porter J, Porter K (2002). Ecological scale and its implications for freshwater fishes in the Florida Everglades. The Everglades, Florida Bay, and Coral Reefs of the Florida Keys: An Ecosystem Sourcebook.

[45] Kushlan JA (1979). Prey choice by tactile-foraging wading birds. Proc. Colon. Waterbird Group.

[46] Gawlik DE, Evans BA, Klassen JA, Gottlieb A, Cyriacks W (2017). Wood Stork use of roadway corridor features in South Florida.

[47] Marzluff JM (2016). A decadal review of urban ornithology and a prospectus for the future. Ibis.

[48] Fuller RA, Warren PH, Gaston KJ (2007). Daytime noise predicts nocturnal singing in urban robins. Biol. Lett..

[49] Schlaepfer MA, Runge MC, Sherman PW (2002). Ecological and evolutionary traps. Trends Ecol. Evol..

[50] Jokimäki J, Huhta E (2000). Artificial nest predation and abundance of birds along an urban gradient. Condor.

[51] Loss SR, Will T, Marra PP (2013). The impact of free-ranging domestic cats on wildlife in the United States. Nat. Commun..

[52] Schmidt KA, Whelan CJ (1999). Effects of exotic *Lonicera* and *Rhamnus* on songbird nest predation. Conserv. Biol..

[53] Borgmann KL, Rodewald AD (2004). Nest predation in an urbanizing landscape: the role of exotic shrubs. Ecol. Appl..

[54] Kahl MP (1972). Comparative ethology of the Ciconiidae. Part 3. The wood storks (genera *Mycteria* and *Ibis*). Ibis.

[55] Frederick PC, Collopy MW (1989). Nesting success of five ciconiiform species in relation to water conditions in the Florida everglades. Auk.

[56] Schoener TW (1968). Sizes of feeding territories among birds. Ecology.

[57] Kushlan JA (1976). Wading bird predation in a seasonally fluctuating pond. Auk.

[58] DeAngelis DL, Trexler JC, Cosner C, Obaza A, Jopp F (2010). Fish population dynamics in a seasonally varying wetland. Ecol. Model..

[59] Anderson WB, Wait DA, Stapp P (2008). Resources from another place and time: responses to pulses in a spatially subsidized system. Ecology.

[60] Williams SE, Shoo LP, Isaac JL, Hoffmann AA, Langham G (2008). Towards an integrated framework for assessing the vulnerability of species to climate change. PLoS Biol..

[61] Lowry H, Lill A, Wong BBM (2013). Behavioural responses of wildlife to urban environments. Biol. Rev..

[62] Wong BBM, Candolin U (2015). Behavioral responses to changing environments. Behav. Ecol..

[63] Sih A (2013). Understanding variation in behavioural responses to human-induced rapid environmental change: a conceptual overview. Anim. Behav..

[64] Snell-Rood EC (2013). An overview of evolutionary causes and consequences of behavioural plasticity. Anim. Behav..

[65] Alberti M (2017). Global urban signatures of phenotypic change in animal and plant populations. Proc. Natl. Acad. Sci..

[66] Hendry AP, Farrugia TJ, Kinnison MT (2008). Human influences on rates of phenotypic change in wild animal populations. Mol. Ecol..

[67] Pomeroy D, Kibuule M (2017). Increasingly urban Marabou Storks start breeding four months early in Kampala, Uganda. Ostrich.

[68] Gilbert NI (2016). Are white storks addicted to junk food? Impacts of landfill use on movement and behaviour of resident white storks (*Ciconia ciconia*) from a partially migratory population. Mov. Ecol..

[69] Thabethe V, Downs CT (2018). Citizen science reveals widespread supplementary feeding of African woolly-necked storks in suburban areas of KwaZulu-Natal, South Africa. Urban Ecosyst..

[70] Martin J, French K, Major R (2010). Population and breeding trends of an urban colonizer: the Australian white ibis. Wildl. Res..

[71] Dorn NJ (2011). Aquatic prey switching and urban foraging by the White Ibis *Eudocimus albus* are determined by wetland hydrologic conditions. Ibis.

[72] Murray MH (2018). From wetland specialist to hand-fed generalist: shifts in diet and condition with provisioning for a recently urbanized wading bird. Philos. Trans. R. Soc. B.

[73] Clergeau P, Yésou P (2006). Behavioural flexibility and numerous potential sources of introduction for the sacred ibis: Causes of concern in western Europe?. Biol. Invasions.

[74] Calle L, Gawlik DE (2011). Anthropogenic food in the diet of the Sacred Ibis (*Threskiornis aethiopicus*), a non-native wading bird in southeastern FL, USA. Fla. Field Nat..

[75] Seto K, Fragkias CM, Güneralp B, Reilly MK (2011). A meta-analysis of global urban land expansion. PLoS ONE.

[76] Seto KC, Güneralp B, Hutrya LR (2012). Global forecasts of urban expansion to 2030 and direct impacts on biodiversity and carbon pools. PNAS.

[77] Gibbs JP (2000). Wetland loss and biodiversity conservation. Conserv. Biol..

[78] Dahl TE (2005). Florida’s wetlands: an update on status and trends 1985 to 1996.

[79] Dahl TE (2011). Status and trends of wetlands in the conterminous United States 2004 to 2009.

[80] Loveless CA (1959). A study of the vegetation in the Florida Everglades. Ecology.

[81] Beerens JM, Noonburg EG, Gawlik DE (2015). Linking dynamic habitat selection with wading bird foraging distribution across resource gradients. PLoS ONE.

[82] Petersen, M. L. Quantifying wading bird resource selection and nesting effort: a tool for the restoration of pulsed ecosystems. Ph.D. Dissertation, Florida Atlantic University (2017).

[83] Telis, P. A. The Everglades Depth Estimation Network (EDEN) for support of ecological and biological assessments. U.S. Geological Survey Fact Sheet: 2006–3087, Reston, Virginia (2006).

[84] Rodgers, J. A. Jr. Protocol for monitoring the reproductive success of Wood Storks in the southeast United States (2005).

[85] Herring G, Gawlik DE (2008). Potential for successful population establishment of the nonindigenous sacred ibis in the Florida Everglades. Biol. Invasions.

[86] Burnham KP, Anderson DR (2004). Multimodel inference understanding AIC and BIC in model selection. Sociol. Methods Res..

[87] R Core Team (2017). R: A language and environment for statistical computing. R version 3.2.4.

[88] Nakagawa S, Shielzeth H (2013). A general and simple method for obtaining R^2^ from generalized linear mixed-effects models. Methods Ecol. Evol..

[89] Shmueli G, Minka TP, Kadane JB, Borle S, Boatwright P (2005). A useful distribution for fitting discrete data: revival of the Conway–Maxell–Poisson distribution. J. R. Stat. Soc. C Appl..

[90] Sellers KF, Shmueli G (2010). A flexible regression model for count data. Ann. Appl. Stat..

[91] Harrison XA (2018). A brief introduction to mixed effects modelling and multi-model inference in ecology. PeerJ.

[92] Brooks ME (2017). “glmmTMB balances speed and flexibility among packages for zero-inflated generalized linear mixed modeling. R J..

[93] Hartig, F. DHARMa: Residual diagnostics for hierarchal (multi-level/mixed) regression models. R package version 0.3.0. https://florianhartig.github.io/DHARMa/ (2020).

[94] Bates D, Mächler M, Bolker BM, Walker SC (2015). Fitting linear mixed-effects models using lme4. J. Stat. Softw..

[95] Clarke KR, Green RH (1988). Statistical design and analysis for ‘biological effects’ study. Mar. Ecol. Prog. Ser..

[96] Anderson MJ (2001). A new method for non-parametric multivariate analysis of variance. Austral Ecol..

[97] Anderson MJ (2006). Distance-based test for homogeneity of multivariate dispersions. Biometrics.

[98] Oksanen, J. *et al*. Vegan: community ecology package https://cran.r-project.org/web/packages/vegan/ (2019).

[99] Clarke KR, Gorley RN (2015). Primer v7: User Manual: Tutorial.

[100] Herring H, Gawlik DE (2011). Resource selection functions for Wood Stork foraging habitat in the southern Everglades. Waterbirds.

[101] Esri. ArcGIS Desktop: Release 10.4.1. Redlands (2015).

