# Supplementary material for: Urban food subsidies reduce natural food limitations and reproductive costs for a wetland bird
Source: Sci Rep. 2020 Aug 20;10:14021. doi: 10.1038/s41598-020-70934-x (PMC7441144; doi:10.1038/s41598-020-70934-x)
Supplement: Supplementary file 1 — Supplementary information. [file 41598_2020_70934_MOESM1_ESM.docx]

Urban food subsidies reduce natural food limitations and reproductive costs for a wetland bird

Authors: Evans, B. A. and D. E. Gawlik

**This file includes:**

Supplementary Methods

Supplementary References

Table S1 to S7

Figures S1 to S9

**Supplementary Methods**

*Productivity and body condition*

In addition to productivity measures, we measured nest success and considered a nest successful if it produced at least one chick to the age of 4 weeks, however, we did not include this measure in our analyses due to too few nests failing during the nestling stage (<5%). Furthermore, we were unable to calculate daily survival rates due to the limited amount of time allowed to visit colonies and to reduce disturbance during the egg laying stage.

We also explored the potential relationship of brood size and clutch size on body condition. Clutch size of Wood Storks in Florida is relatively constant with an average of three eggs ^[1,2,3]^. We minimized disturbance during the egg laying stage, so we did not have a large sample of clutch size data. However, due to asynchronous nest initiations, we were able to determine clutch size for 54 nests. Mean clutch size was 3.44 ± 0.07 se and did not vary significantly across years (ANOVA: F_(2,51)_=1.29, P=0.28; Table S5). This finding is consistent with Herring et al. which found that Great Egret (*Ardea alba*) clutch size did not vary between suboptimal and optimal prey availability years ^[4]^. This consistency in clutch size is likely due to the capacity for brood reduction in wading birds. Several studies have found evidence that food limitation is related to the declines in breeding success and populations of wading birds in the Everglades ^[1,4-9]^.

We also explored the relationship between brood size and body condition of nestlings. Brood size and body condition had a weak positive correlation (Spearman’s Rank Correlation: S=32472, *ρ*=0.26, P=0.041). To confirm that brood size did not greatly influence body condition, we incorporated it into the body condition models. The model containing only brood size, performed worse than all body condition models, including the null model (AICc=75.48). Thus, we did not include brood size as a variable in the body condition models.

*Diet*

Nestlings often regurgitate in the presence of humans, making bolus contents readily available for collection. In the event a targeted nestling did not voluntarily regurgitate, we gently massaged its throat to encourage regurgitation. Once a nestling regurgitated, we left an approximately equal mass of small fish in the nest to compensate for loss of bolus contents. Storks readily consume regurgitated fish from the nest bowl (personal observation) so our collection methods did not reduce the amount of food provided to nestlings.

*Created wetland sampling*

We used two separate trapping methodologies to sample prey in created wetland types to better account for prey availability in sites with diverse water depths and potential prey sizes. We sampled swales and ephemeral ponds with a 1-m^2^ throw-trap. A throw-trap is a 1-m^2^ box with mesh sides and an open top and bottom ^[10]^. We used a 100-cm x 30-cm bar seine to remove aquatic prey from the throw-trap until we had five consecutive sweeps with no additional prey. Throw-traps are the preferred method of sampling fish in shallow, vegetated habitats, such as swales and ephemeral stormwater ponds ^[10]^.

At canals and permanently inundated ponds, we sampled prey using a modified Gee’s G-40 minnow trap (Memphis Net & Twine, Memphis, USA) since throw-traps are not effective in deep water habitats. The maximum size of fish that can be caught in a minnow trap is determined by the size of the funnel opening. Since the Gee’s G-40 minnow trap opening has a diameter of only 2.5-cm, the traps are biased to the capture of small-bodied fish. As such, we modified the opening of the minnow traps to a 10-cm oval, allowing for the capture of larger bodied fish. At each site, we placed an array of minnow traps set at various distances and depths. We placed a minnow trap at opposite edges of the shoreline to capture fish where Wood Storks would most likely forage. In the interior of the pond or canal, we placed three series of equally spaced traps. The number of minnow traps in a series depended on the depth of the canal or pond. For instance, deep canals or ponds (depths > 1.5-m) consisted of three traps: one just below the water surface, one resting on the substrate, and one equal distances between the two traps. For intermediate canal or pond depths (1.0 to 1.5-m), each trap series included two traps: one just below the water surface, and one resting on the substrate. For shallow canal or pond depths (< 1.0-m), each trap series included only one trap resting on the substrate. To allow time for aquatic fauna to enter the traps, we left the minnow traps in place for approximately 24 hrs before we collected and removed trap contents.

**Supplementary References**

1. Kahl, M. P. Jr. Food ecology of the wood stork (*Mycteria americana*) in Florida. *Ecol. Monogr.* **34**, 97-117 (1964).
2. Rodgers, J. A. Jr. Breeding chronology and clutch information for the Wood Stork from museum collections. *J. Field Ornithol.* **61**, 47-53 (1990).
3. Rodgers, J. A. Jr. & Schwikert, S. T. Breeding success and chronology of Wood Storks *Mycteria americana* in northern and central Florida, U.S.A. *Ibis* **139**, 76-91 (1997).
4. Herring, G., Gawlik, D. E., Cook, M. I. & Beerens, J. M. Sensitivity of Great Egrets (*Ardea alba*) and White Ibises (*Eudocimus albus*) to reduced prey availability. *Auk* **127**, 660-670 (2010).
5. Kushlan, J. A. Response of wading birds to seasonally fluctuating water levels: Strategies and their limits. *Colonial Waterbirds* **9**, 155-162 (1986).
6. Kushlan, J. A. & Frohring, P. C. The history of the southern Florida Wood Stork population. *Wilson Bull.* **98**, 368-386 (1986).
7. Frederick, P. C. & Collopy, M. W. The role of predation in determining reproductive success of colonially nesting wading birds in the Florida Everglades. *Condor* **91**, 860-867 (1998).
8. Frederick, P. C. & Spalding, M. G. Factors affecting reproductive success of wading birds (Ciconiiformes) in the Everglades ecosystem. In *Everglades: The Ecosystem and its Restoration* (eds Davis, S. M. & Ogden, J. C.) 659-691 (St. Lucie Press, 1994).
9. Ogden, J. C. A comparison of wading bird nesting dynamics, 1931-1946 and 1974-1989 as an indication of changes in ecosystem conditions in the southern Everglades. In *Everglades: The Ecosystem and its Restoration* (eds Davis, S. M. & Ogden, J. C.) 533-570 (St. Lucie Press, 1994).
10. Jordan, F., Coyne, S. & Trexler, J. C. Sampling fishes in vegetated habitats: effects of habitat structure on sampling characteristics of the 1-m^2^ throw trap. *Trans. Am. Fish. Soc.*, **126**, 1012-1020 (1997).
11. Gawlik, D. E., Petersen, M. L. & Herteux, C. Dry season trophic prey concentrations. Annual report for US Army Corps of Engineers, Jacksonville, Florida (2017).

**Supplemental Tables**

Table S1. Mean productivity and nestling body condition (± se) for storks nesting in urban and natural wetlands across hydrologic conditions, south Florida, 2015-2017. * denotes statistical significance (P<0.05) for pairwise comparisons (urban vs. natural wetland) among hydrologic conditions.

|  | Natural wetland | | | Urban | | | | |
| --- | --- | --- | --- | --- | --- | --- | --- | --- |
|  | Sub | Mod | Opt | Sub | Mod | | Opt | |
| Nests (N) | 12 | 19 | 23 | 38 | | 27 | | 41 |
| Fledglings/nest | 1.17 ± 0.39* | 1.26 ± 0.25* | 2.39 ± 0.22 | 2.00 ± 0.16 | | 2.07 ±0.16 | | 2.58 ± 0.11 |
| Keel score/nest | 2.47 ± 0.10 | -- | 2.74 ± 0.04 | 2.35 ± 0.05 | | -- | | 2.68 ± 0.04 |

Table S2. P values for comparisons of diet breadth across landscape type and hydrologic conditions, south Florida, 2015-2017. Statistically significant P values (P<0.05) are in bold.

|  | UrbSub | NatSub | UrbMod | NatMod | UrbOpt | NatOpt |
| --- | --- | --- | --- | --- | --- | --- |
| UrbSub |  | **0.001** | **0.001** | **0.001** | **0.001** | **0.001** |
| NatSub | **0.000** |  | 0.779 | 0.117 | **0.001** | **0.001** |
| UrbMod | **0.000** | 0.752 |  | **0.014** | **0.001** | **0.001** |
| NatMod | **0.000** | 0.094 | **0.013** |  | **0.001** | **0.001** |
| UrbOpt | **0.000** | **0.000** | **0.000** | **0.000** |  | 0.724 |
| NatOpt | **0.000** | **0.000** | **0.000** | **0.000** | 0.718 |  |

Landscape type: Urb=urban, Nat=natural wetland. Hydrologic condition: Sub=suboptimal, Mod=moderate, and Opt=optimal.

Table S3. Values of ANOSIM R statistic across diet samples, using created wetlands as the explanatory variable. A value close to 1, implies separation among samples whereas a value close to 0 implies similarity.

|  | Natural wetland | | | Urban | | |
| --- | --- | --- | --- | --- | --- | --- |
|  | Sub | Mod | Opt | Sub | Mod | Opt |
| R statistic | 0.123 | 0.399 | 0.364 | 0.121 | 0.278 | 0.313 |
| P value | 0.02 | 0.001 | 0.001 | 0.11 | 0.001 | 0.001 |

Hydrologic condition: Sub=suboptimal, Mod=moderate, and Opt=optimal.

Table S4. Mean length (± se) (Kruskal-Wallis test, χ^2^=351.60, P<0.001, df=5) and weight (± se) (Kruskal-Wallis test, χ^2^=343.95, P<0.001, df=5) of prey items found within nestling stork boluses in urban and natural wetlands across hydrologic conditions, south Florida, 2015-2017. Letters denote statistical significance (P<0.05) using Dunn’s method for pairwise comparisons.

|  | Natural wetland | | | | | Urban | | | |
| --- | --- | --- | --- | --- | --- | --- | --- | --- | --- |
|  | Sub | Mod | | Opt | | Sub | Mod | Opt | |
| Boluses (N) | 66 | | 78 | 100 | 137 | | 156 | | 106 |
| Length (cm) (± sem) | 4.10 ± 0.04^c^ | | 5.45 ± 0.11^a^ | 5.50 ± 0.13^a^ | 4.12 ± 0.09^d^ | | 5.28 ± 0.10^b^ | | 5.74 ± 0.15 ^a^ |
| Weight (g) (± sem) | 2.16 ± 0.11^c^ | | 7.01 ± 0.49^a^ | 8.64 ± 0.75^a^ | 3.83 ± 0.27^c^ | | 8.58 ± 0.50^b^ | | 9.12 ± 0.77^a^ |

Hydrologic condition: Sub=suboptimal, Mod=moderate, and Opt=optimal.

Table S5. Mean clutch size (± se) for storks nesting in south Florida, 2015-2017.

|  | | 2015 (moderate) | | 2016 (suboptimal) | 2017 (optimal) | |
| --- | --- | --- | --- | --- | --- | --- |
| Nests (N) | 7 | | 17 | | | 30 |
| Clutch size | 3.14 ± 0.14 | | 3.47 ± 0.26 | | | 3.50 ± 0.33 |

Table S6. Percent biomass (percent frequency) of species identified within stork boluses, south Florida, 2015-2017. N=number of boluses collected from each colony.

|  | Percent biomass (Percent frequency) | | | | | |
| --- | --- | --- | --- | --- | --- | --- |
| Prey species | Suboptimal  Urban | Suboptimal  Natural | Moderate  Urban | Moderate  Natural | Optimal  Urban | Optimal  Natural |
| **Large-bodied native fish** |  |  |  |  |  |  |
| Centrarchidae |  |  |  |  |  |  |
| *Enneacanthus gloriosus* | 1 (2) | -- | 1 (5) | 1 (2) | 1 (2) | 1 (3) |
| *Lepomis gulosus* | 41 (17) | 13 (5) | 31 (16) | 18 (10) | 42 (27) | 39 (11) |
| *Lepomis macrochirus* | 4 (4) | -- | 1 (1) | <1 (1) | 1 (1) | -- |
| *Lepomis marginatus* | 10 (7) | 8 (4) | 2 (7) | 4 (11) | 1 (2) | 4 (8) |
| *Lepomis microlophus* | 3 (2) | 1 (<1) | 6 (2) | -- | <1 (<1) | 2 (2) |
| *Lepomis punctatus* | 2 (1) | 1 (<1) | 25 (14) | 42 (28) | 31 (34) | 43 (42) |
| *Micropterus salmoides* | 1 (2) | -- | 8 (2) | -- | 3 (6) | <1 (1) |
| Clupeidae |  |  |  |  |  |  |
| *Dorosoma petenense* | -- | -- | <1 (<1) | -- | 1 (1) | -- |
| Esocidae |  |  |  |  |  |  |
| *Esox americanus* | -- | -- | -- | <1 (<1) | <1 (<1) | <1 (<1) |
| *Esox niger* | <1(<1) | <1 (<1) | -- | -- | <1 (<1) | -- |
| Ictaluridae |  |  |  |  |  |  |
| *Ameiurus natalis* | <1 (<1) | -- | -- | -- | -- | <1 (<1) |
| *Ameiurus nebulosus* | <1 (1) | <1 (<1) | 3 (2) | 13 (5) | 1 (<1) | -- |
| *Noturus gyrinus* | -- | <1 (<1) | -- | <1 (2) | -- | <1 (1) |
|  |  |  |  |  |  |  |
| **Non-native fish** |  |  |  |  |  |  |
| Callichthyidae |  |  |  |  |  |  |
| *Hoplosternum littorale* | <1 (<1) | -- | -- | -- | -- | -- |
| Cichlidae |  |  |  |  |  |  |
| *Cichla ocellaris* | -- | -- | -- | -- | -- | 2 (<1) |
| *Cichlasoma bimaculatum* | 2 (2) | <1 (<1) | <1 (<1) | 1 (1) | 1 (1) | <1 (<1) |
| *Cichlasoma urophthalmus* | 2 (1) | 3 (1) | 2 (2) | 2 (3) | < 1 (2) | <1 (1) |
| *Hemichromis letourneuxi* | <1 (<1) | 55 (60) | <1 (<1) | 7 (17) | <1 (1) | 3 (9) |
| *Oreochromis aureus* | 1 (<1) | -- | <1 (1) | -- | 9 (2) | 1 (1) |
| *Parachromis managuense* | -- | -- | -- | -- | 3 (<1) | -- |
| *Pelmatolapia mariae* | <1 (<1) | <1 (<1) | -- | <1 (1) | <1 (1) | 3 (<1) |
| Claridae |  |  |  |  |  |  |
| *Clarias batrachus* | -- | 2 (<1) | -- | -- | 1 (<1) | -- |
| Cyprinidae |  |  |  |  |  |  |
| *Ctenopharyngodon idella* | 3 (1) | 1 (<1) | 8 (1) | 7 (2) | 4 (<1) | -- |
| Poeciliidae |  |  |  |  |  |  |
| *Belonesox belizanus* | -- | 2 (1) | -- | -- | -- | -- |
|  |  |  |  |  |  |  |
| **Small native fish** |  |  |  |  |  |  |
| Cyprinodontidae |  |  |  |  |  |  |
| *Cyprinodon variegatus* | -- | <1 (<1) | -- | -- | <1 (<1) | <1 (<1) |
| *Jordanella floridae* | 1 (3) | 1 (4) | 1 (5) | <1 (4) | <1 (1) | <1 (1) |
| Fundulidae |  |  |  |  |  |  |
| *Fundulus confluentus* | -- | 5 (13) | 1 (5) | <1 (1) | -- | <1 (1) |
| *Lucania goodei* | <1 (3) | <1 (<1) | <1 (1) | -- | <1 (2) | <1 (1) |
| Percidae |  |  |  |  |  |  |
| *Gambusia holbrooki* | 3 (30) | 1 (3) | <1 (10) | <1 (4) | <1 (3) | <1 (8) |
| *Heterandria formosa* | <1 (<1) | -- | <1 (<1) | -- | <1 (1) | -- |
| *Poecilia latipinna* | 1 (3) | 3 (3) | 1 (8) | 2 (7) | 1 (5) | 1 (5) |
|  |  |  |  |  |  |  |
| **Crayfish species** |  |  |  |  |  |  |
| *Procambarus* spp. | 6 (7) | 1 (<1) | 1 (3) | <1 (1) | <1 (2) | <1 (1) |
| **Amphibian species** |  |  |  |  |  |  |
| *Anura* spp. | 2 (4) | -- | <1 (1) | <1 (<1) | -- | -- |
| *Siren* spp. | 4 (<1) | -- | <1 (<1) | 2 (<1) | -- | -- |
|  |  |  |  |  |  |  |
| **Trash** |  |  |  |  |  |  |
| Trash | 11 (3) | -- | 5 (1) | -- | <1 (1) | -- |
| **N** | **137** | **66** | **156** | **78** | **106** | **100** |

Table S7. A priori model hypotheses predicting body condition and productivity of storks in south Florida, 2015-2017. Landscape type = urban or natural wetland, Hydrologic condition = suboptimal, moderate, or optimal.

| Hypothesis | Model |
| --- | --- |
| Global^*^ | Y = Landscape type + Hydrologic condition + Landscape type*Hydrologic condition |
| Landscape type^*^ | Y = Landscape type |
| Hydrologic condition^*^ | Y = Hydrologic condition |
| Landscape type/Hydrologic condition | Y = Landscape type + Hydrologic condition |
| Null | Y = Colony |

Y = Number of chicks fledged per nest

Y = Average keel score per nest

^*^Colony added as random variable to all models

**Supplementary Figures**


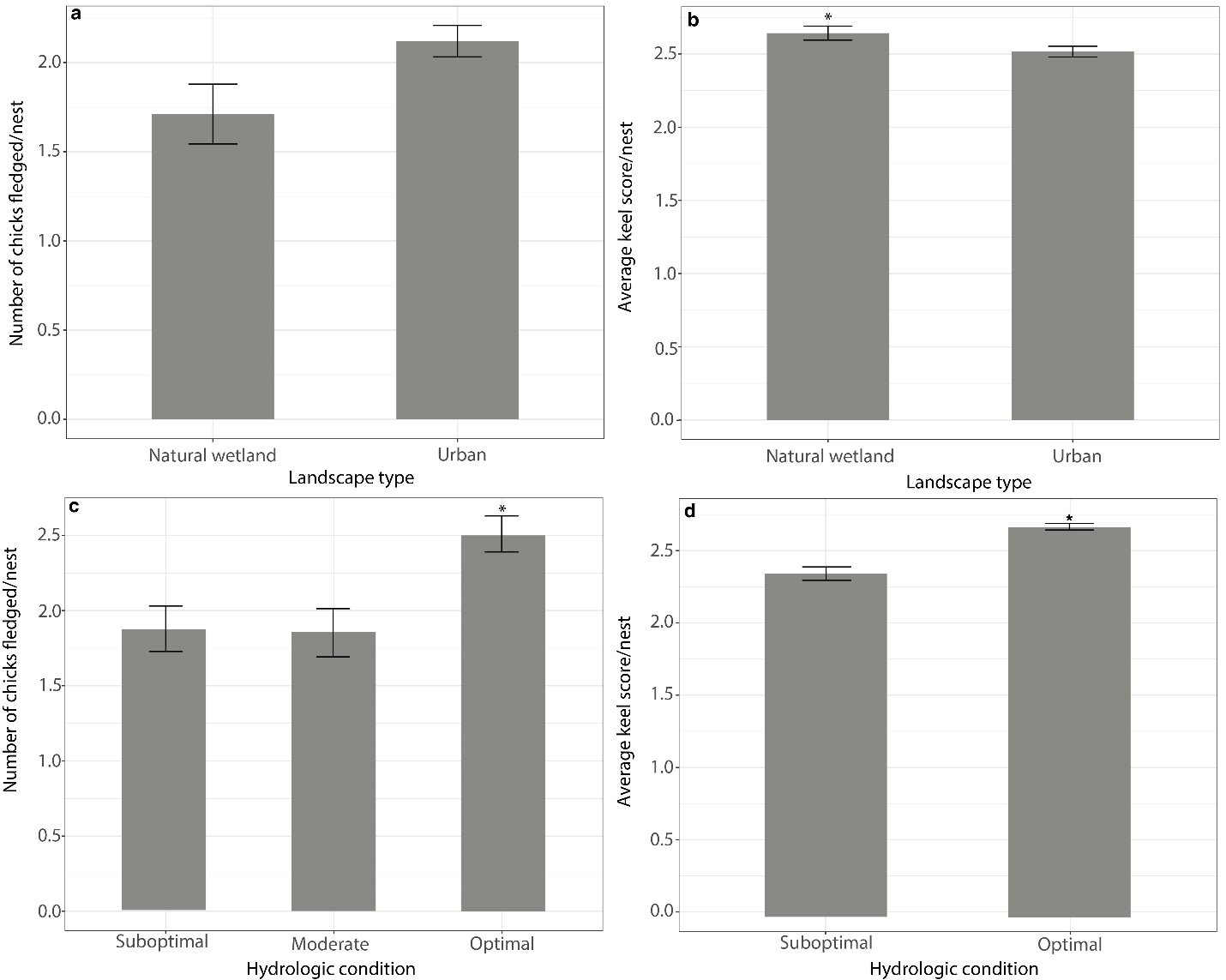


Figure S1. Mean number of chicks fledged per nest (± se) across: a) landscape type and c) hydrologic conditions, south Florida, 2015-2017. Mean keel score per nest (± se) across b) landscape type and d) hydrologic conditions, south Florida, 2015-2017. * denote statistical significance (P<0.05) using Dunn’s method for pairwise comparisons.


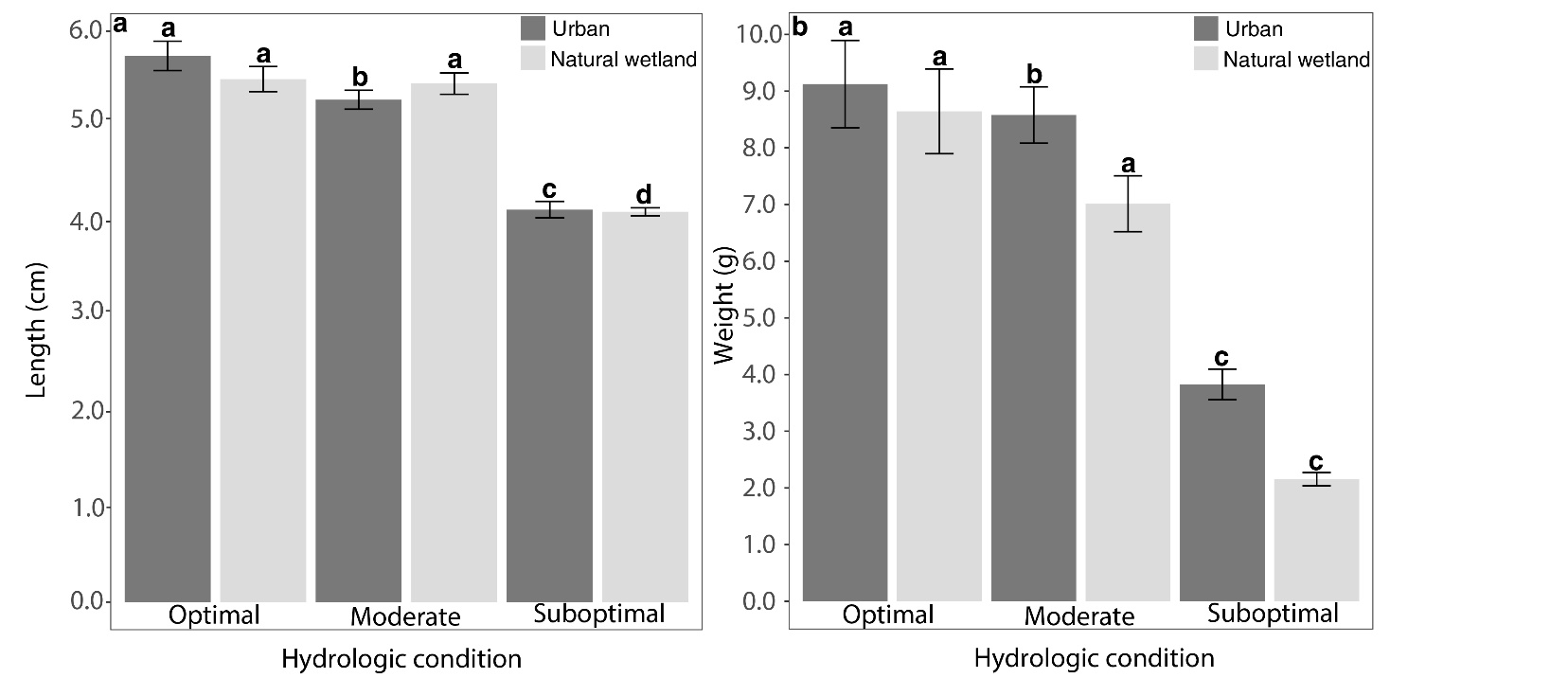


Figure S2. a) Mean length (cm ± se) and b) weight (g ± se) of prey found within nestling stork boluses across landscape type and hydrologic conditions, south Florida, 2015-2017. Letters denote statistical significance (P<0.05) using Dunn’s method for pairwise comparisons.


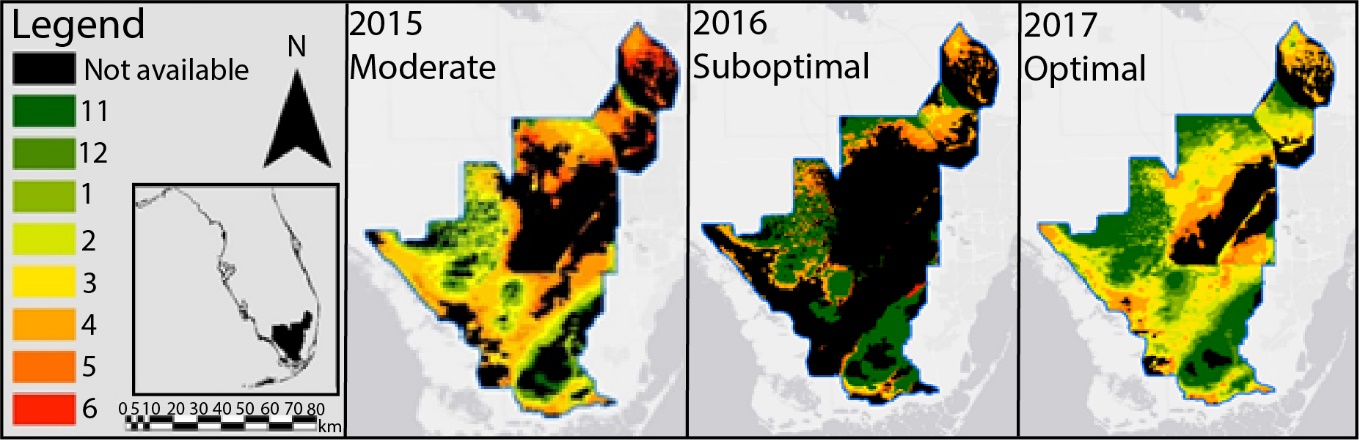


Figure S3. Monthly available habitat in Everglades system. Colors indicate the month that habitat became available for stork foraging. Black indicates that the habitat did not become available. November (11) and December (12) on each map represents previous calendar year (adapted from ^[11]^).


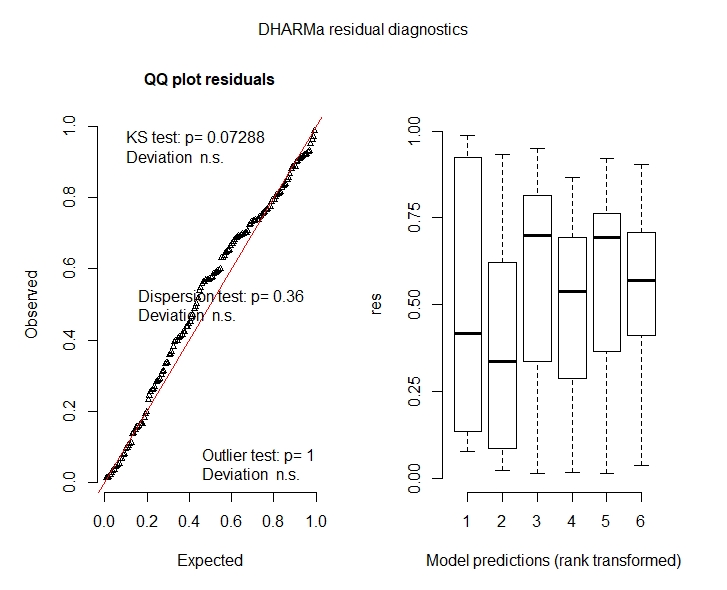


Figure S4. Model diagnostics for Conway-Maxwell-Poisson distributed productivity model. Left panel: Q-Q plot to detect overall deviations from expected distribution. Includes tests for uniformity, dispersion, and outliers. KS (Kolmogorov-Smirnov) test D=0.10173, P=0.07288; Dispersion test = 0.92606, P=0.36; outlier test: tests if there are more simulation outliers than expected, P=1.0. Right panel: Residuals vs. predicted values. The boxplot of the residuals vs. model predictions assess the homogeneity of variances and identify outliers (no outliers were identified).


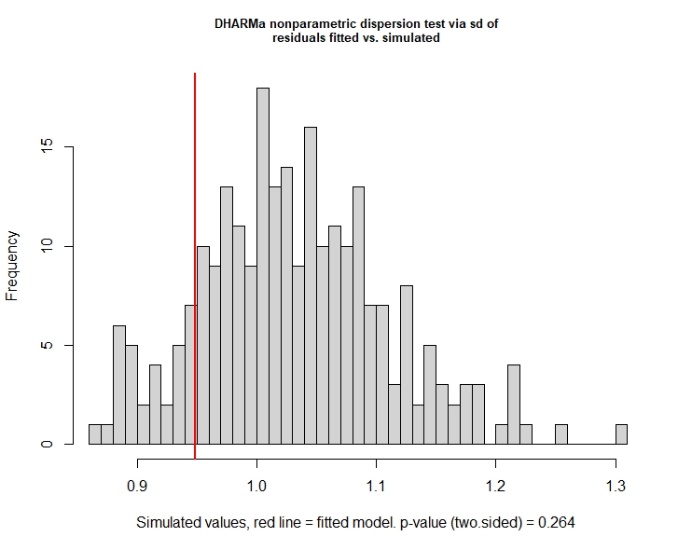


Figure S5. Model diagnostics for Conway-Maxwell-Poisson distributed productivity model. Histogram of model residuals.


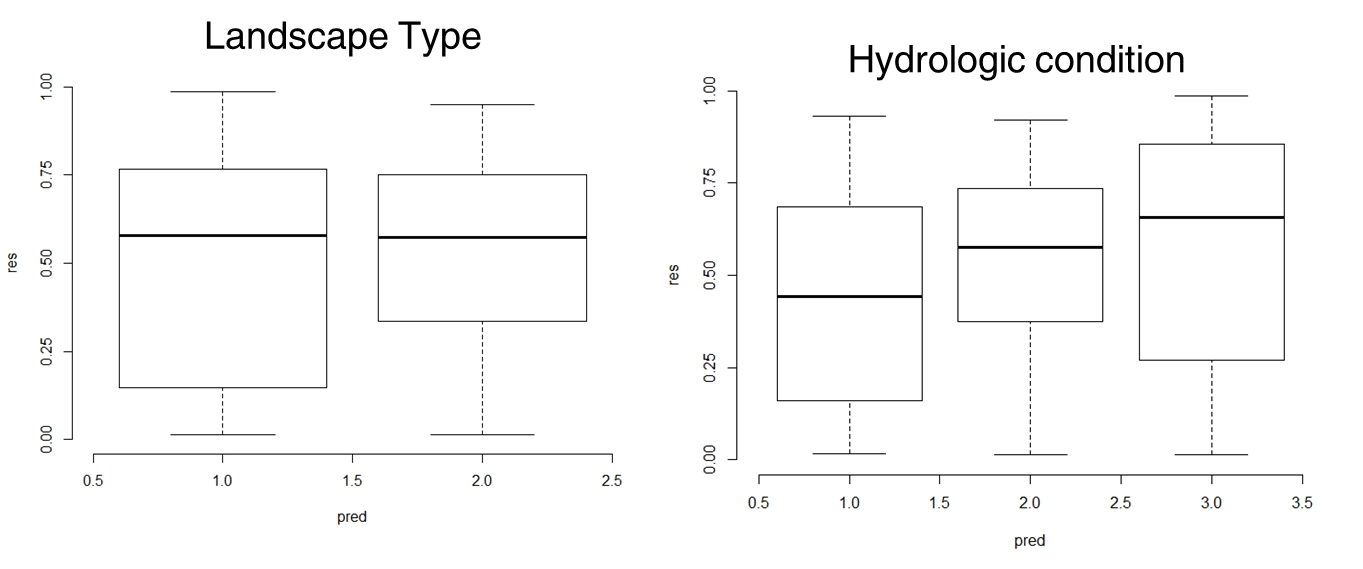


Figure S6. Model diagnostics for Conway-Maxwell-Poisson distributed productivity model. Boxplot of residuals against categorical predictors. The boxplot of the residuals vs. predictors assess the homogeneity of variances and identify outliers (no outliers were identified).


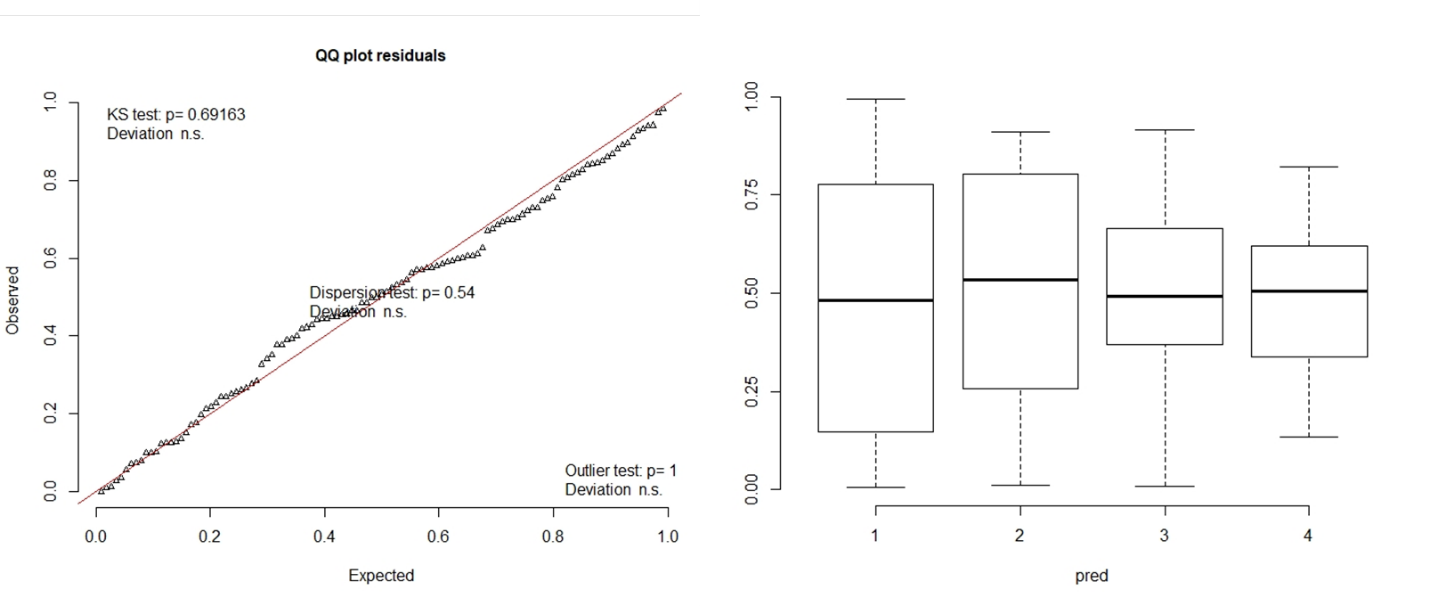


Figure S7. Model diagnostics for Gaussian distributed body condition model. Left panel: Q-Q plot to detect overall deviations from expected distribution. Includes tests for uniformity, dispersion, and outliers. KS (Kolmogorov-Smirnov) test D=0.066954, P=0.69193; Dispersion test = 0.94155, P=0.54; outlier test: tests if there are more simulation outliers than expected, P=1.0. Right panel: Residuals vs. predicted values. The boxplot of the residuals vs. model predictions assess the homogeneity of variances and identify outliers (no outliers were identified).


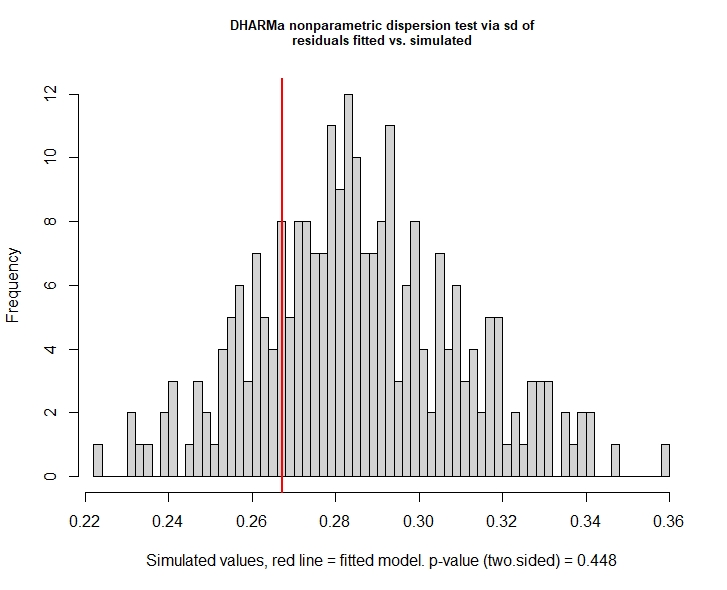


Figure S8. Model diagnostics for Gaussian distributed body condition model. Histogram of model residuals.


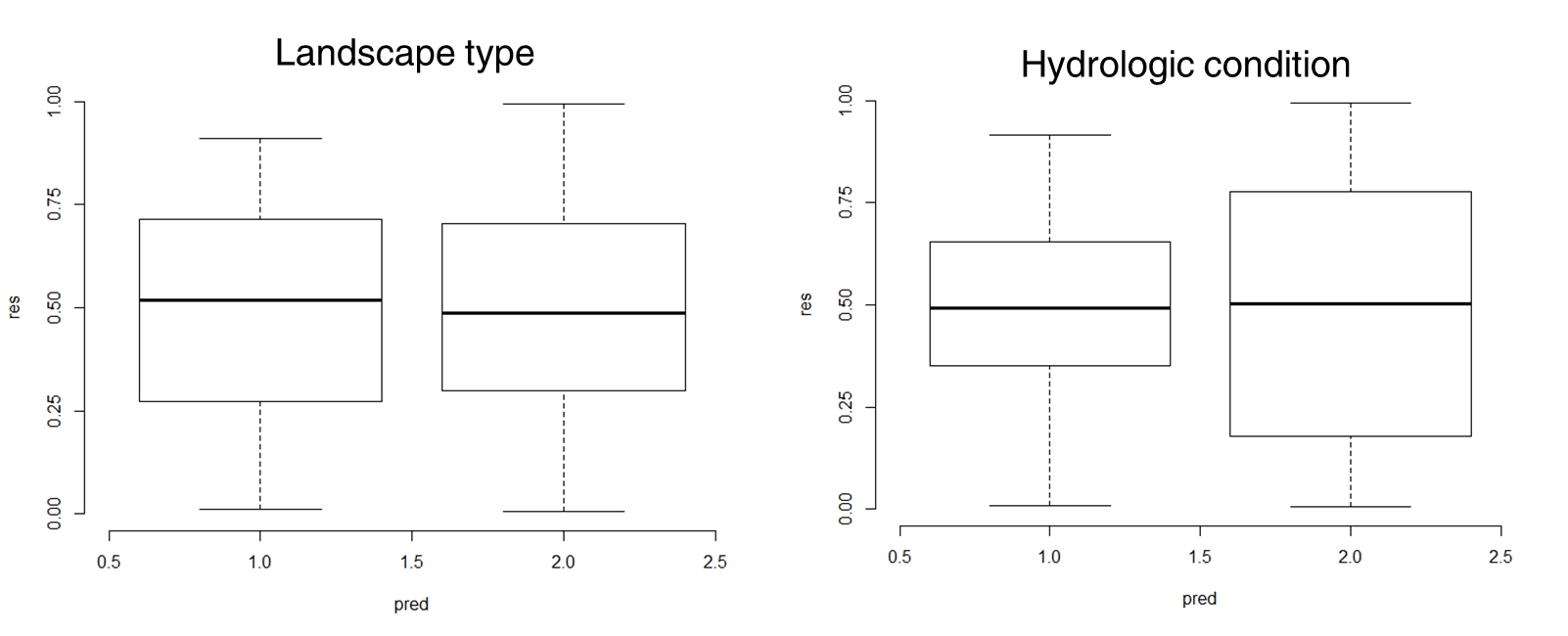
Figure S9. Model diagnostics for Gaussian distributed body condition model. Boxplot of residuals against categorical predictors. The boxplot of the residuals vs. predictors assess the homogeneity of variances and identify outliers (no outliers were identified).
